# Design of hypoxia responsive CRISPR-Cas9 for target gene regulation

**DOI:** 10.1038/s41598-023-43711-9

**Published:** 2023-10-05

**Authors:** Yan An, Chandana S. Talwar, Kwang-Hyun Park, Woo-Chan Ahn, Su-Jin Lee, Seong-Ryeong Go, Jin Hwa Cho, Do Yon Kim, Yong-Sam Kim, Sayeon Cho, Jeong-Hoon Kim, Tae-Jip Kim, Eui-Jeon Woo

**Affiliations:** 1 Division of Biomedical Research, Korea Research Institute of Bioscience and Biotechnology, Daejeon, 305-333 Republic of Korea; 2 Department of Bioscience, University of Science and Technology, Daejeon, 305-333 Republic of Korea; 3 Division of Animal, Horticultural and Food Sciences, Graduate School of Chungbuk National University, Cheongju, 28644 Republic of Korea; 4 Laboratory of Molecular and Pharmacological Cell Biology, College of Pharmacy, Chung-Ang University, Seoul, 06974 Republic of Korea

**Keywords:** Proteins, Gene therapy

## Abstract

The CRISPR–Cas9 system is a widely used gene-editing tool, offering unprecedented opportunities for treating various diseases. Controlling Cas9/dCas9 activity at specific location and time to avoid undesirable effects is very important. Here, we report a conditionally active CRISPR–Cas9 system that regulates target gene expression upon sensing cellular environmental change. We conjugated the oxygen-sensing transcription activation domain (TAD) of hypoxia-inducing factor (HIF-1α) with the Cas9/dCas9 protein. The Cas9-TAD conjugate significantly increased endogenous target gene cleavage under hypoxic conditions compared with that under normoxic conditions, whereas the dCas9-TAD conjugate upregulated endogenous gene transcription. Furthermore, the conjugate system effectively downregulated the expression of SNAIL, an essential gene in cancer metastasis, and upregulated the expression of the tumour-related genes HNF4 and NEUROD1 under hypoxic conditions. Since hypoxia is closely associated with cancer, the hypoxia-dependent Cas9/dCas9 system is a novel addition to the molecular tool kit that functions in response to cellular signals and has potential application for gene therapeutics.

## Introduction

CRISPR–Cas9 gene editing technology represents a revolutionary breakthrough in genetic engineering, offering a promising platform for improving the treatment of various genetic and infectious diseases^1–4^. Owing to its simple design and powerful ability to edit different loci simultaneously, the CRISPR–Cas9 system has been extensively developed and customized since its discovery. However, its inability to precisely edit genes in specific tissues or cells within certain time frames may result in undesirable consequences^5^. Hence, various efforts to broaden the CRISPR–Cas9 tool kit through conditional control of its activity have been reported. Some of the recent advances include small molecule-induced activation of Cas9^6–9^, recruitment of transcriptional modulators to catalytically dead Cas9 (dCas9)^10,11^, photochemically caged Cas9^12^, and NIR- controlled release of Cas9^13^. Alternatively, optical control of gRNA has also been reported by several independent groups^14–16^; these groups used a similar strategy that masked the spacer region and regulated release by light-induced photolysis^17^. However, most of these studies have mainly focused on modulating the function of Cas9 by external stimulation, and very few studies have reported the exploitation of the internal physiological environment of cells to directly control CRISPR–Cas9 activity. Given the complexity of the biological system and the unknown cytotoxic effects or tissue penetration of external stimuli, controlling CRISPR–Cas9 function based on homeostatic changes in the cellular environment would be very important and have potential application in the biomedical field^18^. In this study, we used one such condition, hypoxia, to act as an internal stimulus to control the CRISPR–Cas9 system.

Currently, extensive research on the application of the CRISPR/Cas9 system to cancer treatment has been reported. For instance, the expression of E-cadherin, Cyclin-Dependent Kinase Inhibitor 1A (p21) and human Bcl-2-associated X protein (hBax) in bladder urothelial carcinoma cells, the silencing of endogenous Cyclin-Dependent Kinase 11 (CDK11) gene expression in osteosarcoma cell line, and the knockout of the SHC binding and spindle associated 1 (SHCBP1) gene in breast cancer cells using the CRISPR/Cas9 system have proven effective in reducing cancer progression^19–21^. Furthermore, the catalytic mutant of the Cas9 protein, termed dCas9, is effective in facilitating epigenetic changes in several genes associated with cancer, such as Granulin (GRN) in liver cancer, Phosphatase and Tensin Homolog (PTEN) in breast cancer, Suppressor of activator protein 1 (SARI) in colon cancer and Homeobox A11 (HOXA11) in myelogenous leukaemia, resulting in reduced cancer progression^22–25^. However, the gene-editing function of the conventional CRISPR–Cas9 system is not specific to cancer cells and thus can result in undesirable side effects in normal cells. Hence, the conditional regulation of CRISPR–Cas9 function based on the physiological environment of cells is of great importance for cancer treatment.

Herein, for the first time, we report the design of a hypoxia-regulated CRISPR/Cas9 system that acts as a protein switch to mediate target gene regulation in response to hypoxic changes in the cellular environment. The underlying idea is to combine the oxygen-sensing capability of Hypoxia inducible factor 1- subunit alpha (HIF-1α), a master regulatory protein that senses hypoxic conditions in cells, with the gene cleavage ability of Cas9 or the gene activation ability of dCas9 to develop a novel hypoxia- dependent CRISPR–Cas9 system. HIF-1α is a transcription factor that responds to changes in oxygen concentrations in the cellular environment. Under normoxic conditions, HIF-1α is tightly regulated by a set of enzymes (Prolyl Hydroxylase Domain -PHD) that hydroxylate the oxygen-dependent degradation domain (ODD) of HIF-1α at specific residues, namely, P402 and P564^26,27^. The hydroxylated HIF-1α subunits are recognized by the von-Hippel Lindau (VHL) protein and targeted for degradation by the proteasome pathway^28^. Under hypoxic conditions, no hydroxylation of these key residues occurs, which allows the protein to function as a transcription factor to upregulate the expression of various target genes in response to hypoxia^26^. In vitro, as little as three hours of hypoxia is sufficient to stabilize the HIF-1α protein, which is involved in cell proliferation, invasion, angiogenesis and metastasis of cancer cells^29^. Herein, we designed a hypoxia-sensitive CRISPR–Cas9/dCas9 system by conjugating the oxygen-sensitive domain of HIF-1α to the effector Cas9/dCas9 protein and analyzed the functional activity of this conjugate system and its potential application in cancer therapy.

## Results

### Selection of the hypoxia-sensing domain of HIF-1α

**Figure 1:**
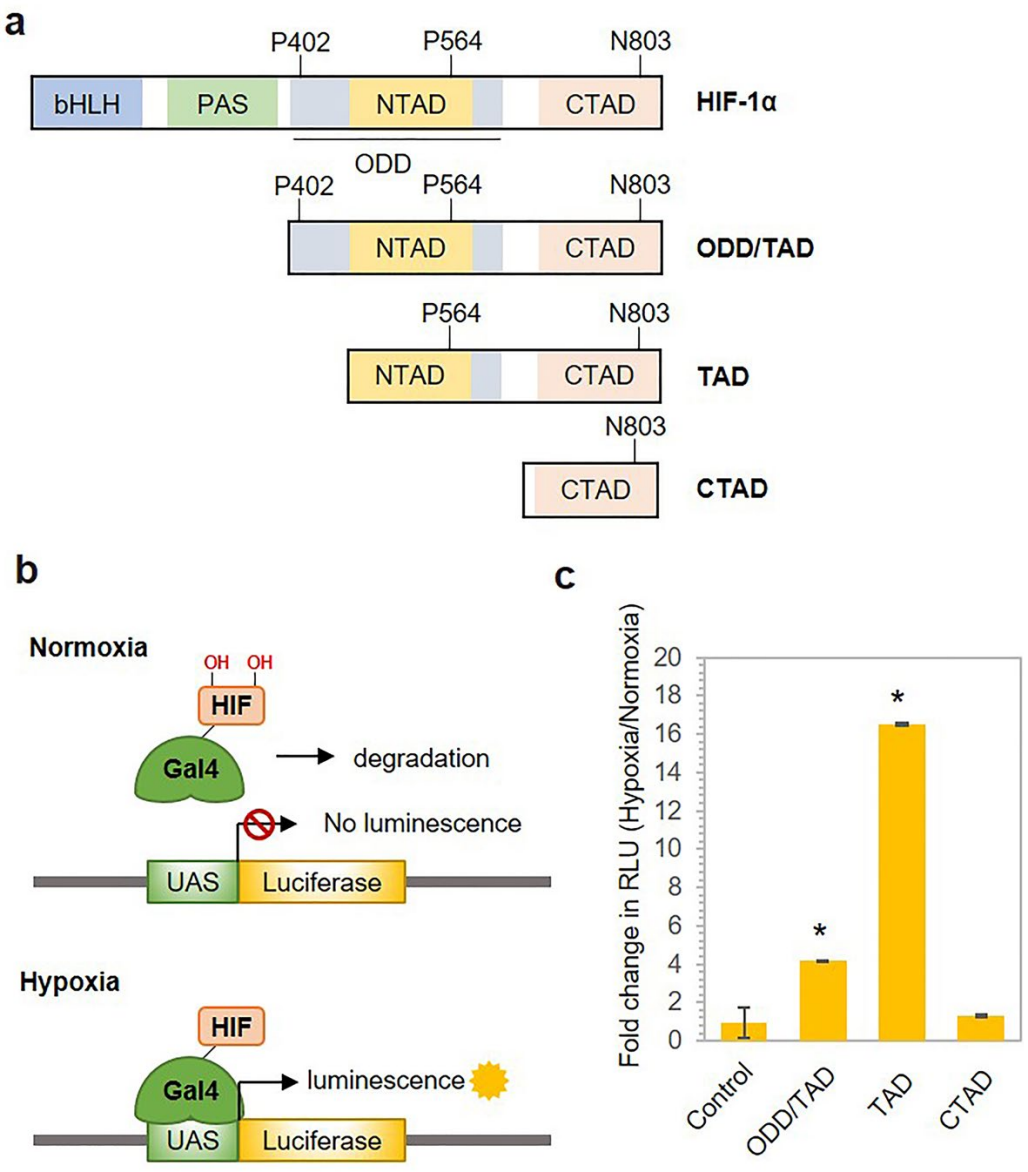
Selection of the hypoxia-sensing domain of HIF-1α. (**a**) Schematic diagram showing the domain distribution of HIF-1α. Residues marked in the diagram represent the key residues involved in sensing hypoxia. (**b**) Graphical representation showing the principle of the luciferase reporter assay. (**c**) Bar graph showing the hypoxia-sensing efficiency of the truncated variants of HIF-1α as examined with a luciferase reporter assay. The Y-axis shows the fold change in the relative luminescence units (RLUs) under hypoxic conditions normalized to those obtained under normoxic conditions. The error bars represent the standard deviation (SD) of the results from three technical replicates. P values were calculated using two-tailed t test. *P < 0.05, **P < 0.01 and ***P < 0.001 vs. the control group.

Previous studies have shown the response of the HIF-1α protein to low-oxygen conditions and its role in sensing hypoxia at the cellular level^26,27^. However, HIF-1α as a whole is not a favourable option for conjugation with the CRISPR/Cas9 system due to its size and DNA-binding function in transcription. Hence, we sought to define a region of HIF-1α that would confer hypoxia- dependent stability to the conjugate protein. Since the residues P402, P564 and N803 are essential for sensing oxygen and for the subsequent degradation of the HIF-1α protein, we generated three truncated variants of the HIF-1α protein, namely, oxygen-dependent degradation domain/Transactivation domain (ODD/TAD: aa 390–826), Transactivation domain (TAD: aa 531–826) and C-terminal Transactivation domain (CTAD: aa 786–826), which contain these key residues, as shown in Fig. 1a^26^. To determine hypoxia-sensing activity of these variants, we conjugated each variant to a Galactose utilization positive control protein 4 (Gal4) binding domain and analyzed them with a luciferase reporter system. The pGL4.21-Gal4-Luc plasmid contains the GAL4 upstream activator sequence (UAS) upstream of the luciferase reporter gene, and the Gal4-binding domain binds to the UAS and activates the downstream gene inside the cell^30^ (Fig. 1b). Upon transfection of the reporter plasmid and vectors encoding the HIF-1α variants into HEK293 cells, one set of cells was treated with CoCl_2_ to imitate hypoxic conditions, whereas the other set of transfected cells was grown in standard cell culture conditions, which are referred to here as normoxic conditions. After 48 h of transfection, the luciferase activity was measured in both sets of cells and reported as relative luminescence units (RLUs). We observed that the cultures grown under hypoxic conditions showed higher luminescence than the cultures grown under normoxic conditions. This increase in luminescence was observed in cells expressing all three HIF-1α variants. The increased luminescence is expressed as the fold change in comparison to the luminescence of the cells grown under normoxic conditions, as shown in Fig. 1c. The results showed that of all three HIF-1α variants, the TAD exhibited the highest difference in luminescence, with a fold change of 16.5, while the ODD/TAD and the CTAD resulted in 4.5-and 1.3-fold increases in RLUs upon hypoxic conditions, respectively. In parallel, we evaluated the corresponding protein levels of the three constructs via western blotting assay (Supplementary Fig. S1a,b). We observed that the ODD/TAD and TAD proteins were expressed at high levels under hypoxic conditions than under normoxic conditions. However, we did not observe any hypoxia-dependent increase in CTAD protein expression. As our final aim was to develop a hypoxia-sensitive Cas9/dCas9 system capable of regulating gene expression, we decided to use the TAD domain, which exhibited the highest fold change, for to sense hypoxia in our further design.

### Construction of a hypoxia-dependent Cas9 regulator by conjugation with TAD

**Figure 2:**
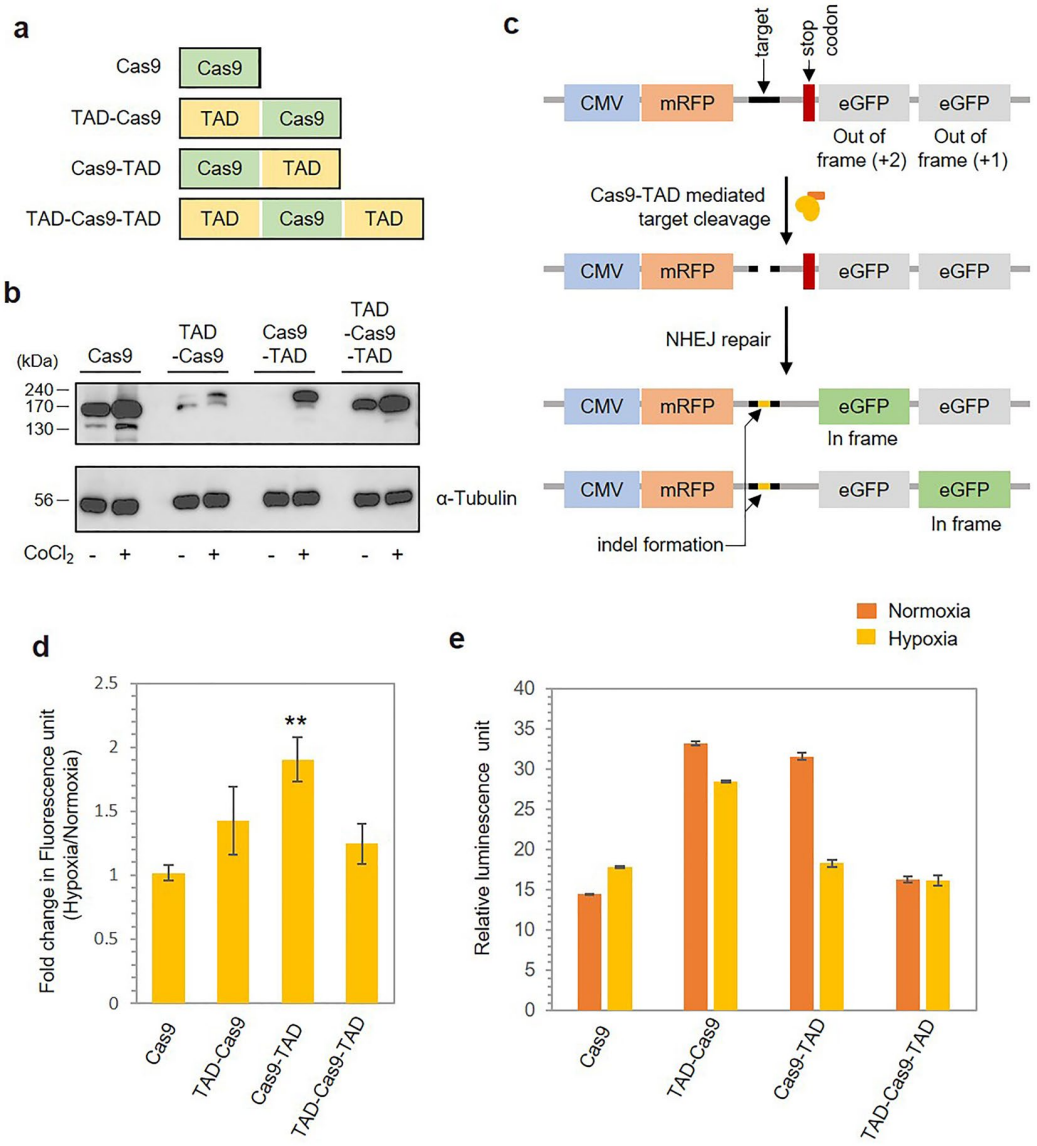
Construction of a hypoxia-dependent Cas9 regulator by conjugation with TAD. (**a**) Schematic presentation of the conjugate proteins created by the fusion of the TAD and Cas9. The diagrams shows the placement of the TAD with respect to Cas9 in each of the three variants constructed. (**b**) Western blotting results showing the protein expression of the conjugate proteins under normoxic and hypoxic conditions. The protein expression was investigated using anti-Cas9 antibodies 12 h after transfection. The lower lane shows the protein level of α-Tubulin. (**c**) Schematic representation of the mRFP-eGFP reporter assay used to investigate the hypoxia-sensitive target cleavage activity of the conjugate proteins. (**d**) Bar graph showing the quantified results from the mRFP-eGFP reporter assay. The fluorescence intensity was quantified using ImageJ software and is expressed as the fold change in fluorescence normalized to the fluorescence observed under normoxic conditions. The error bars represent the standard deviation (SD) of the results from three technical replicates. P values were calculated using two-tailed t test. *P < 0.05, **P < 0.01 and ***P < 0.001 vs. the Cas9 group. (**e**) Hypoxia-dependent Cas9-mediated cleavage of the target luciferase gene was assessed with a luciferase reporter assay. The marked value indicates the luciferase activity observed under hypoxic conditions normalized to that observed under normoxic conditions. The error bars represent the standard deviation (SD) of two technical replicates.

Next, we conjugated TAD to the Cas9 protein to analyze its effect on target gene cleavage under hypoxic conditions. Depending on its spatial location in the conjugate form, the position of TAD with respect to that of Cas9 might be important for sensing hypoxia as well as for the activity of Cas9. Hence, we constructed three variants of conjugate proteins, namely, TAD-Cas9, Cas9-TAD and TAD-Cas9-TAD, by placings the TAD region either at the N-terminus, the C-terminus or both termini of the Cas9 protein, respectively, as shown in Fig. 2a. First, we investigated the hypoxia-dependent stability of the conjugate proteins through western blotting. After 12 h of hypoxic treatment, the intensity of the bands corresponding to the conjugate proteins was much higher under hypoxic conditions than under normoxic conditions when probed with an anti-cas9 antibody. The Cas9 or tubulin protein levels did not exhibit any hypoxia-dependent changes, suggesting that observed change was dependent on the TAD (Fig. 2b). Quantification of the band intensities revealed 1.6-fold higher protein levels of TAD-Cas9, 2.6-fold higher protein levels of Cas9-TAD and 1.8-fold higher protein levels of TAD-Cas9-TAD under hypoxic conditions, suggesting the potential involvement of the TAD in maintaining the stability of the conjugate proteins (Supplementary Fig. S2a). Additionally, this experiment was performed in a true hypoxic condition in a hypoxic chamber with 1% O_2_, where the observed results closely mirrored those observed under CoCl_2_-induced hypoxia (Supplementary Fig. S2b). We observed higher levels of Cas9-TAD under hypoxic conditions compared to normoxic conditions. However, there was no significant increase in levels for either Cas9 or TAD-Cas9-TAD under hypoxic conditions. We were unable to detect a strong visible band for TAD-Cas9 in both hypoxic and normoxic environments. This absence of a clear signal persisted even in the presence of CoCl_2_-induced hypoxia albeit with some visibility.

Next, we examined the hypoxia-dependent cleavage activity of the Cas9 and TAD conjugates using the mRFP-eGFP reporter system. In this system, two eGFP-coding sequences are inserted after a target site in such a way that either (3n + 1) or (3n + 2) nucleotide insertions or deletions at the target sequence lead to eGFP expression. In the absence of target cleavage, cells express only mRFP due to the out-of-frame arrangement of the two eGFP genes. If a Cas9-mediated double-strand break occurs at the target site, the concurrent indels result in frameshift mutations of the downstream sequence, causing at least one of the eGFP genes to be expressed (Fig. 2c)^31^. To analyze the effect of hypoxia on the conjugate proteins, we used the Cas9 protein in the absence of the TAD as a negative control. We transfected the mRFP-eGFP reporter system along with the plasmids encoding the Cas9 conjugate variants and a plasmid encoding sgRNA specific for the target site into HEK293 cells and cultured the cells for 48 h under hypoxic and normoxic conditions. We measured the eGFP activity and mRFP activity in the cultures grown under both conditions. Red fluorescence was used as the internal control to normalize the observed green fluorescence. For all three conjugate variants, the cultures grown under hypoxic conditions showed higher fluorescence than those grown under normoxic conditions. We quantified the observed fluorescence and calculated the fold change in intensity between the hypoxic and normoxic groups (Fig. 2d and Supplementary Fig. S3). For the Cas9 protein, we did not observe any significant change in fluorescence intensity between the hypoxic and normoxic conditions. However, Cas9-TAD exhibited a significant increase in fluorescence under hypoxic conditions, corresponding to a 1.9-fold increase, whereas TAD-Cas9 and TAD-Cas9-TAD showed marginal changes of 1.4-and 1.2-fold increases in fluorescence under hypoxic conditions, respectively. Since higher GFP expression reflects the formation of indels by cleavage of the target DNA, this result suggests the higher cleavage activity of Cas9-TAD under hypoxia.

We further examined the ability to cleave the target gene using the luciferase reporter system in which the target is the luciferase gene inserted in the plasmid. The luciferase gene in the pGL4.21-Gal4-Luc plasmid was targeted for cleavage by Cas9 in the conjugate proteins using sgRNA. The effect of hypoxia on the abilities of the conjugate proteins to cleave the luciferase gene was assessed by measuring the luminescence of the cultures. For all three conjugate proteins, we observed reduced luminescence in the cultures grown under hypoxic conditions compared to that of the cultures grown under normoxic conditions. The results showed a significantly lower luminescence intensity of Cas9-TAD under hypoxic conditions. The Cas9-TAD and TAD-Cas9 constructs resulted in 42% and 15% reductions in luciferase activity, respectively, under hypoxic conditions compared with normoxic conditions, but TAD-Cas9-TAD did not show any difference (Fig. 2e). For the Cas9 protein without a TAD conjugate, we did not observe any significant loss of luciferase activity between the hypoxic and normoxic groups. Consistent with the fluorescence reporter assay, Cas9-TAD shows higher efficiency of hypoxia-dependent downregulation of target gene expression than the other two conjugate proteins. Hence, we selected Cas9-TAD for the cleavage of endogenous target genes in cells.

### Hypoxia-dependent regulation of endogenous gene expression by Cas9-TAD

**Figure 3:**
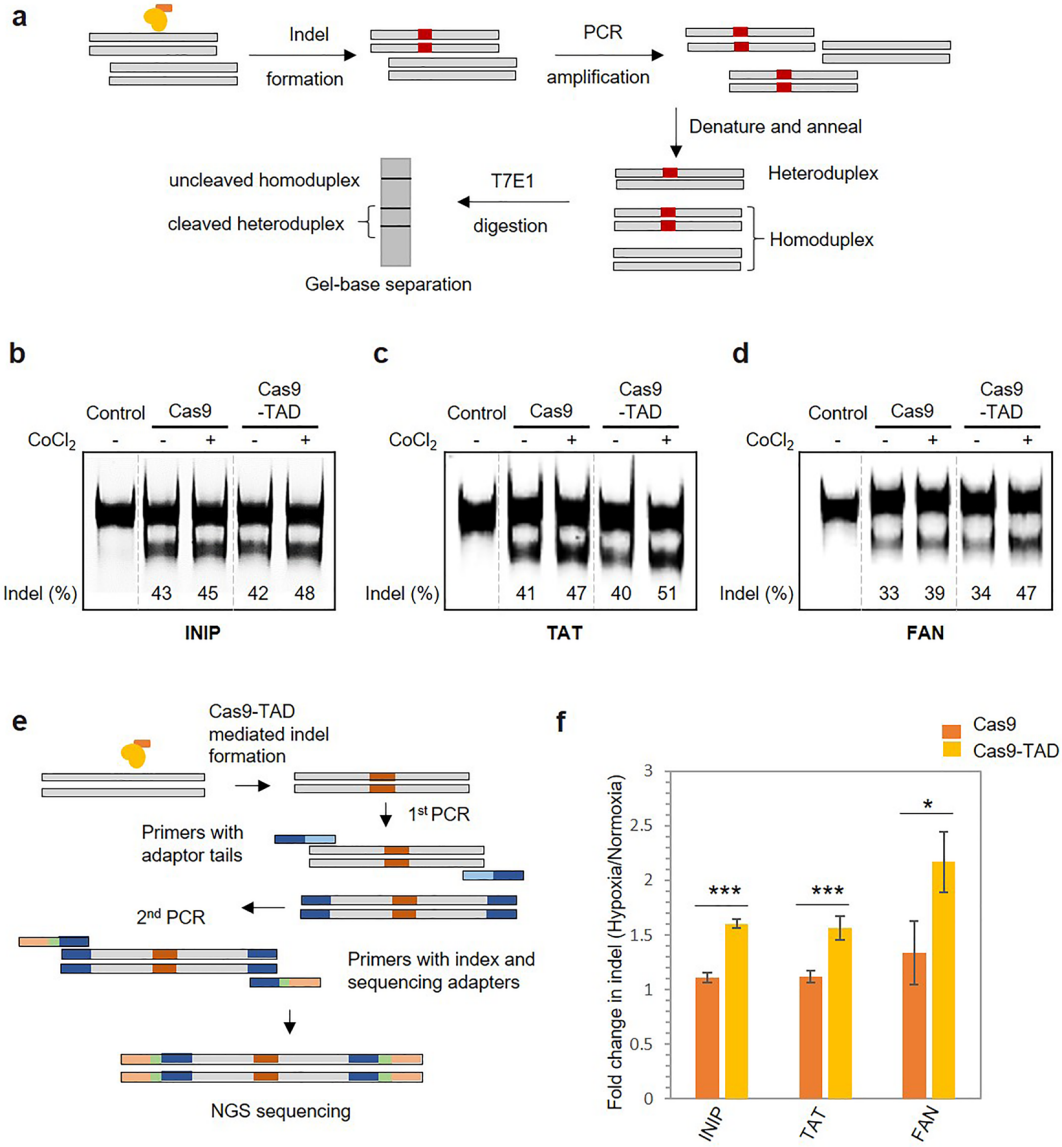
Hypoxia-dependent regulation of endogenous gene expression by Cas9-TAD. (**a**) Schematic representation of the T7 endonuclease assay. Cas9-TAD activity on the endogenous genes (**b**) INIP, (**c**) TAT, and (**d**) FAN as shown by the T7 endonuclease assay. The gel images are shown. Different parts of the same gel are grouped together to place Cas9 and Ca9-TAD conditions next to each other. Original gel image can be found in Supplementary data (Supplementary Fig. S10a–c). Representative gel image of T7 endonuclease-treated PCR products amplified from the target sites. (−) and (+) represent the presence and absence of cobalt chloride, respectively. The intensity of the bands from the T7 endonuclease assay was quantified using ImageJ software, and the percentage of indels was calculated as the ratio of the intensity of the cleaved fragments to the sum of the intensities of the cleaved and uncleaved products and is shown below each lane. (**e**) Schematic presentation of the steps involved in NGS sequencing. (**f**) Cas9-TAD activity on the endogenous genes INIP, TAT and FAN as shown by NGS sequencing. The graph shows the fold change in indel formation. The plotted values indicate the average of the results, and the error bars represent the standard deviation of the results from three technical replicates. *P* values were calculated using two-tailed t test. ^*^P < 0.05, **P < 0.01 and ***P < 0.001 vs. the Cas9 group.

We investigated whether the hypoxia-dependent cleavage activity of Cas9-TAD can effectively work on genes located in genomic DNA. We selected three clinically relevant genes, namely, INTS3 And NABP Interacting Protein (INIP), Tyrosine Aminotransferase (TAT) and FANCD2/FANCI-associated nuclease 1 (FAN), as targets. The INIP gene is a key player in cervical incompetence and superficial mycosis^32^, while TAT is associated with tyrosinemia disorder^33^ and FAN is associated with Fanconi anaemia, which is a recessive autosomal disorder^34,35^. First, we examined target gene cleavage by Cas9-TAD using a T7 endonuclease assay (T7E1 assay). In this assay, the nucleotide region containing the target locus is amplified by PCR; then, any heteroduplex structure that was formed by mismatch after Cas9-mediated cleavage and subsequent DNA repair in the cell is digested, producing two DNA fragments smaller than the size of the intact nucleotide sequence (Fig. 3a)^36^. Based on the region targeted by Cas9-TAD, each of the genes should yield smaller fragments of the following sizes: 144 bp and 143 bp for INIP, 145 bp and 133 bp for TAT, and two 149-bp fragments for FAN. After transfection of the plasmid carrying Cas9-TAD, the cells were treated with CoCl_2_ and harvested for comparative analysis after 48 h. For all three target genes, slightly higher intensities of the fragmented bands were observed under hypoxic conditions than under normoxic conditions. We quantified the intensities of the bands and calculated the percentage of indel formation as a ratio of the intensity of the cleaved fragments to the sum of the intensities of the cleaved and uncleaved products. As shown in Fig. 3b–d and Supplementary Fig. S10a–c, we observed slightly increased indel formation at the target sites of all three genes under hypoxic conditions. At the INIP target site, Cas9-TAD showed 6% higher indel formation under hypoxic conditions than under normoxic conditions (Fig. 3b). The difference was more significant for the TAT and FAN genes, in which 11% and 13% higher indel formation was observed under hypoxic conditions, respectively, compared to the 6% higher indel formation observed in the case of Cas9 (Fig. 3c,d). Although significant Cas9-TAD-mediated cleavage was observed under normoxic conditions, it was likely due to the high amount of Cas9-TAD plasmid transfected into the cell; 1.5 µg plasmid per 1 × 10^6^ cells was required to visually observe the cleavage pattern. The T7 endonuclease assay is dependent on visual observation of indels by PAGE and is often affected by the length and identity of base pair mismatches, flanking sequences and secondary structures of DNA^36^.

Hence, for accurate analysis, we further examined Cas9-mediated cleavage of endogenous genes through next-generation sequencing (NGS). After transfection and hypoxic treatment for 48 h, the target site was amplified using a two-step PCR process and analyzed using targeted NGS (Fig. 3e). We used the same concentration of the Cas9-TAD plasmid for the NGS cleavage assay, and the change in indel formation under hypoxic conditions was expressed as the fold change compared to that under normoxic conditions (Fig. 3f). The results showed more significant changes in target gene cleavage under hypoxic conditions. Relative to normoxic conditions, hypoxic conditions resulted in 1.6-fold, 1.6-fold and 2.3-fold higher indel formation for INIP, TAT and FAN, respectively, in the presence of Cas9-TAD, whereas no significant change was observed in the presence of the Cas9 protein alone. We also performed NGS analysis of the three target genes using two other conjugate protein variants, namely, TAD-Cas9 and TAD-Cas9-TAD, from the previous section. Relative to normoxic conditions, hypoxic conditions resulted in slight increases in TAD-Cas9 activity (1.2-fold, 1.4-fold and 1.2-fold changes in the cleavage of the INIP, FAN and TAT genes, respectively), whereas TAD-Cas9-TAD did not show any significant difference between hypoxic and normoxic conditions (Supplementary Fig. S4). The NGS result of a significantly higher cleavage rate under hypoxic conditions clearly demonstrate that Cas9-TAD can be used to regulate endogenous target gene cleavage under hypoxic conditions.

### Construction of a hypoxia-dependent dCas9 regulator by conjugation with TAD

**Figure 4:**
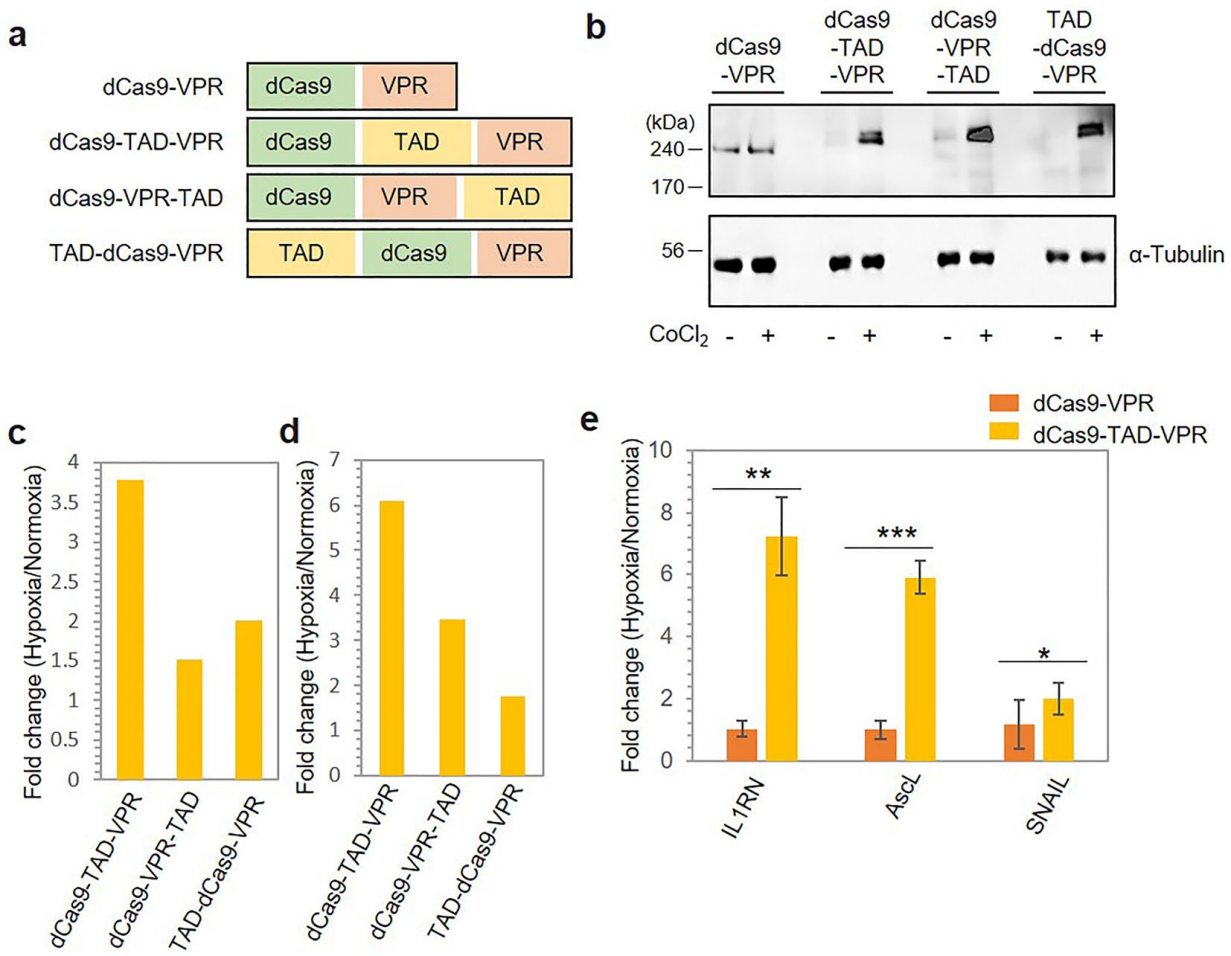
Construction of a hypoxia-dependent regulator by conjugation of dCas9 with TAD. (**a**) Schematic presentation of the conjugate proteins created by the fusion of TAD, dCas9 and VPR. (**b**) Western blotting results showing the protein expression of the conjugate proteins under normoxic and hypoxic conditions. The protein expression was investigated using anti-Cas9 antibodies 12 h after transfection. (**c**) The luciferase gene located on a plasmid under a GAL4 promoter was targeted using sgRNA, and the luciferase activity is presented as RLUs. The fold change in RLUs is shown after normalization to the RLUs observed under normoxic conditions. (**d**) Hypoxia-dependent transcriptional activation of the target luciferase gene expressed from a genomic locus. (**e**) dCas9-TAD-VPR activity on the endogenous genes IL1RN, AscL and SNAIL as shown by qPCR. The fold change in the mRNA level is plotted on the y-axis. The standard deviation was calculated from the results of three replicates. P values were calculated using two-tailed t test. *P < 0.05, **P < 0.01 and ***P < 0.001 vs. the dCas9-VPR group.

dCas9, a catalytically inactive Cas9 mutant that lacks endonuclease activity, is widely used for the transcriptional regulation of target genes in conjugation with transcriptional activators or repressors^37,38^. A previous report showed that dCas9 conjugated with the small domain activator VPR (VP64-p65-Rta) effectively activated target gene transcription^37^. Therefore, we decided to conjugate the TAD to the dCas9-VPR protein to achieve hypoxia-sensitive transcriptional regulation. Although the TAD itself contains regions (531–575 and 786–826) that bind to transcription factors^39^, our preliminary experiment showed that transcriptional activation by dCas9 and the TAD alone was negligible (Supplementary Fig. S5). To optimize the spatial arrangement with respect to dCas9 and VPR, three conjugate protein variants with varying positions of TAD were constructed, as shown in Fig. 4a. First, we examined their corresponding protein levels under hypoxic and normoxic conditions by western blotting. After hypoxic treatment, the protein levels were examined using an anti-Cas9 antibody used to detect dCas9 (Fig. 4b). dCas9-VPR did not show any observable difference in protein expression, whereas the other three constructs containing TAD showed higher protein expression under hypoxic conditions than under normoxic conditions, with 1.25-fold, 1.7-fold and 5.4-fold higher protein levels of dCas9-TAD-VPR, dCas9-VPR-TAD and TAD-dCas9-VPR, respectively, under hypoxic conditions (Supplementary Fig. S6a). In the case of dCas9-VPR, we observed overall less intensity for the bands in both hypoxic and normoxic conditions. We performed a luciferase reporter assay in the presence of MG132, a proteasome inhibitor^40^, and all three conjugate proteins showed increased luminescence in the presence of the proteasome inhibitor compared to that without the inhibitor (Supplementary Fig. S6b), suggesting the involvement of protein degradation. Since HIF-1α is known to be degraded in an oxygen- and ubiquitin/proteasome-dependent manner^41,42^, the hypoxia-sensitive change in the system is more likely driven by ubiquitin-mediated degradation due to the TAD^43^.

Next, we investigated the ability of the conjugate proteins to activate hypoxia-sensitive transcription by targeting the promoter of the luciferase gene in the plasmid. In the assay system, the upstream Gal4 promoter region in the pGL4.21-Gal4-Luc plasmid, which contains four binding site repeats, was targeted using one sgRNA, and upon binding of the conjugate protein to the GAL4 promoter, the downstream luciferase gene was expressed. Under hypoxic conditions, we observed increased luciferase activity compared to that under normoxic conditions for all three variants, as shown in Fig. 4c. The dCas9-TAD-VPR construct produced the highest fold change in luminescence under hypoxic conditions (3.8-fold), whereas dCas9-VPR-TAD and TAD-dCas9-VPR yielded 1.5- and 2.0-fold increases under hypoxic conditions, respectively. We further examined the transcriptional activation of genes located in genomic DNA in stable HEK293 cell lines expressing the luciferase gene under the control of the GAL4 promoter. Consistent with the luciferase reporter assay, dCas9-TAD-VPR showed a significant 6.1-fold change in luminescence under hypoxic conditions, while dCas9-VPR-TAD and TAD-dCas9-VPR also showed 3.5- and 1.7-fold changes in luminescence, respectively (Fig. 4d). The cumulative results suggest that dCas9-TAD-VPR exhibits the highest hypoxia-sensitive transcriptional activation of target genes compared with the other two conjugate protein variants.

Next, we investigated the ability of dCas9-TAD-VPR to target a promoter of an endogenous gene located in genomic DNA. We selected three clinically relevant genes, namely, Interleukin 1 receptor antagonist (IL1RN), Achaete-scute homologue 1 (AscL1) and Zinc finger protein SNAI1 (SNAIL), as target genes. IL1RN is one of the key players in immune and inflammatory responses^44,45^. AscL1 plays a role in neuronal commitment and differentiation and is essential for the generation of olfactory and autonomic neurons^46,47^. SNAIL is associated with cancer progression^48,49^. To compare the relative changes in transcription, we used dCas9-VPR, with the absence of the TAD, as a control. Previously, targeting multiple sites in a promoter region was shown to be effective for dCas9-mediated transcriptional activation^50^. Therefore, we targeted multiple binding sites in the promoters (four sites in the IL1RN promoter and the AscL promoter and two sites in the SNAIL promoter) by sgRNA mixtures. After 48 h of hypoxic treatment, the cells were harvested, and the indicated mRNA levels were quantified through qPCR to measure target gene upregulation. For all three target genes, we observed a significant increase in the mRNA levels under hypoxic conditions compared with those under normoxic conditions (Fig. 4e). The fold change in the mRNA levels was calculated relative to that observed under normoxic conditions, and the results showed that the dCas9-TAD-VPR construct produced 7.7-fold higher expression of IL1RN and sixfold and 1.7-fold higher expression of AscL and SNAIL, respectively, under hypoxic conditions. However, dCas9-VPR did not cause any change in target gene transcription under hypoxic conditions compared to normoxic conditions. The low expression of SNAIL might have partly resulted from the fewer binding sites (only two) in its promoter compared with the four sites in the other two gene promoters. Additionally, we performed qPCR analysis of the three target genes using the other two conjugated protein variants: dCas9-VPR-TAD and TAD-dCas9-VPR. Although the expression of dCas9-VPR- TAD resulted in 1.6-fold higher AscL1 mRNA levels under hypoxic conditions, we did not observe any significant change in the IL1RN and SNAIL mRNA levels. The expression of TAD-dCas9-VPR also showed a similar pattern, with 1.9-fold higher AscL1 mRNA levels under hypoxic conditions, whereas no significant change was observed in the IL1RN and SNAIL mRNA levels (Supplementary Fig. S7). Additionally, to consolidate our observations we assessed mRNA levels of IL1RN in actual hypoxic condition targeted by dCas9-TAD-VPR. Observed results closely aligned with that of CoCl_2_-induced hypoxia with an 8.7-fold increase in hypoxic condition as compared to normoxic condition (Supplementary Fig. S8c,d). Taken together, the results clearly indicate that dCas9- TAD-VPR is able to upregulate the expression of endogenous target genes with multiple binding sites in their promoters in a hypoxia-dependent manner.

### Hypoxia-dependent regulation of cancer target gene expression by Cas9/dCas9 gene regulators

**Figure 5:**
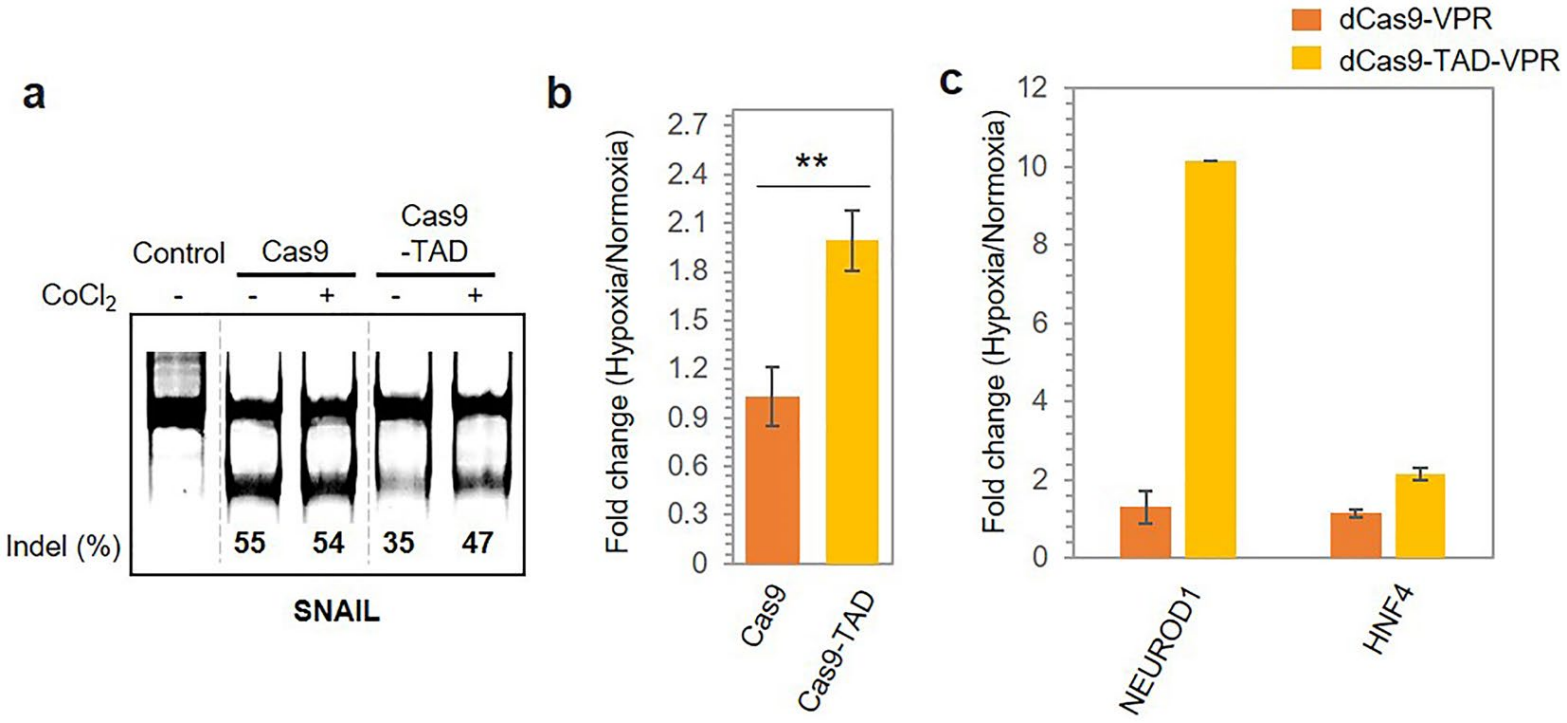
Hypoxia-dependent modulation of cancer target gene expression by Cas9/dCas9 gene regulators. (**a**) Cas9-TAD mediated the downregulation of SNAIL expression, as shown by the T7 endonuclease assay. Different parts of the same gel are grouped together to place Cas9 and Ca9-TAD conditions next to each other. Original gel image is shown in supplementary data (Supplementary Fig. S10d). (**b**) Cas9-TAD activity on the endogenous gene SNAIL as shown by NGS sequencing. The error bars represent the standard deviation of the results from three technical replicates. P values were calculated using two-tailed t test. *P < 0.05, **P < 0.01 and ***P < 0.001 vs. the Cas9 group. (**c**) dCas9-VPR-TAD mediated the upregulation of the expression of the endogenous genes NEUROD 1 and HNF4 as shown by an qPCR assay. The standard deviation was calculated from the results of two replicates.

A growing body of work has shown the association of the SNAIL, Neuronal Differentiation 1 (NEUROD1) and Hepatocyte nuclear factor 4 (HNF4) genes with cancer. Overexpression of SNAIL promotes epithelial-mesenchymal transition and thereby drives cancer metastasis, whereas ectopic expression of NEUROD1 and HNF4 is shown to reduce the progression of medulloblastoma and prostate cancer^48,49,51–53^. Since modulating the expression of these genes is important for cancer therapy, we examined the possibility of downregulating SNAIL expression and upregulating NEUROD1 and HNF4 expression in a hypoxia-dependent manner using Cas9/dCas9 gene regulators. First, we targeted SNAIL using sgRNA and assessed the cleavage of this gene under hypoxic conditions by Cas9-TAD using the T7 endonuclease assay. We observed 12% higher cleavage of SNAIL by Cas9-TAD under hypoxic conditions compared to normoxic conditions, whereas Cas9 without the TAD only showed a marginal change in indel formation (Fig. 5a). NGS sequencing further supported these results; Cas9-TAD resulted in 1.9-fold higher indel formation under hypoxic conditions, whereas Cas9 did not show any significant change in activity (Fig. 5b). We further evaluated SNAIL downregulation using the two other conjugate protein variants from the previous section, and TAD-Cas9 and TAD-Cas9-TAD resulted in 1.6-fold and 1.5-fold higher disruption of SNAIL under hypoxic conditions, respectively, as per NGS sequencing (Supplementary Fig. S8a). Nevertheless, the observed fold difference for TAD-Cas9 and TAD-Cas9-TAD was less than that for Cas9-TAD, which was consistent with our previous results.

Next, we investigated the hypoxia-dependent upregulation of NEUROD1 and HNF4 expression using dCas9-TAD-VPR. For the NEUROD1 gene, we targeted multiple binding sites in its promoter region with four different sgRNAs to ensure higher transcriptional activation. For the HNF4 gene, we targeted only one site in its promoter using the corresponding sgRNA. When the fold change in mRNA expression due to dCas9-TAD-VPR activity was compared with that due to dCas9-VPR activity, we observed an increase in the mRNA levels of both target genes in the presence of dCas9-TAD-VPR in a hypoxia-dependent fashion, as shown in Fig. 5c. Notably, NEUROD1 mRNA was expressed at tenfold higher levels under hypoxic conditions than under normoxic conditions, whereas HNF4 mRNA was expressed at twofold higher levels. We did not observe any significant upregulation of target gene expression by dCas9-VPR under hypoxic conditions, and equal mRNA levels were observed under both conditions. When we investigated the change in NEUROD1 and HNF4 expression using dCas9-VPR-TAD and TAD-dCas9-VPR, no significant difference was observed between hypoxic and normoxic conditions (Supplementary Fig. S8b). Furthermore, to validate our observations, we undertook an assessment of the NEUROD1 expression profile under genuine hypoxic conditions, while employing the dCas9-TAD-VPR construct. We observed a 7.4-fold increase in mRNA level upon hypoxic treatment which is comparable to that observed in CoCl_2_-induced hypoxia (Supplementary Fig. S8c,d). This result suggests that the conjugate Cas9/dCas9 system developed in this study could modulate the expression of various target genes related to cancer. Using Cas9 and dCas9 alternatively, we demonstrated that the system is capable of downregulating and upregulating target gene expression, which has potential application in cancer therapy.

## Discussion

**Figure 6:**
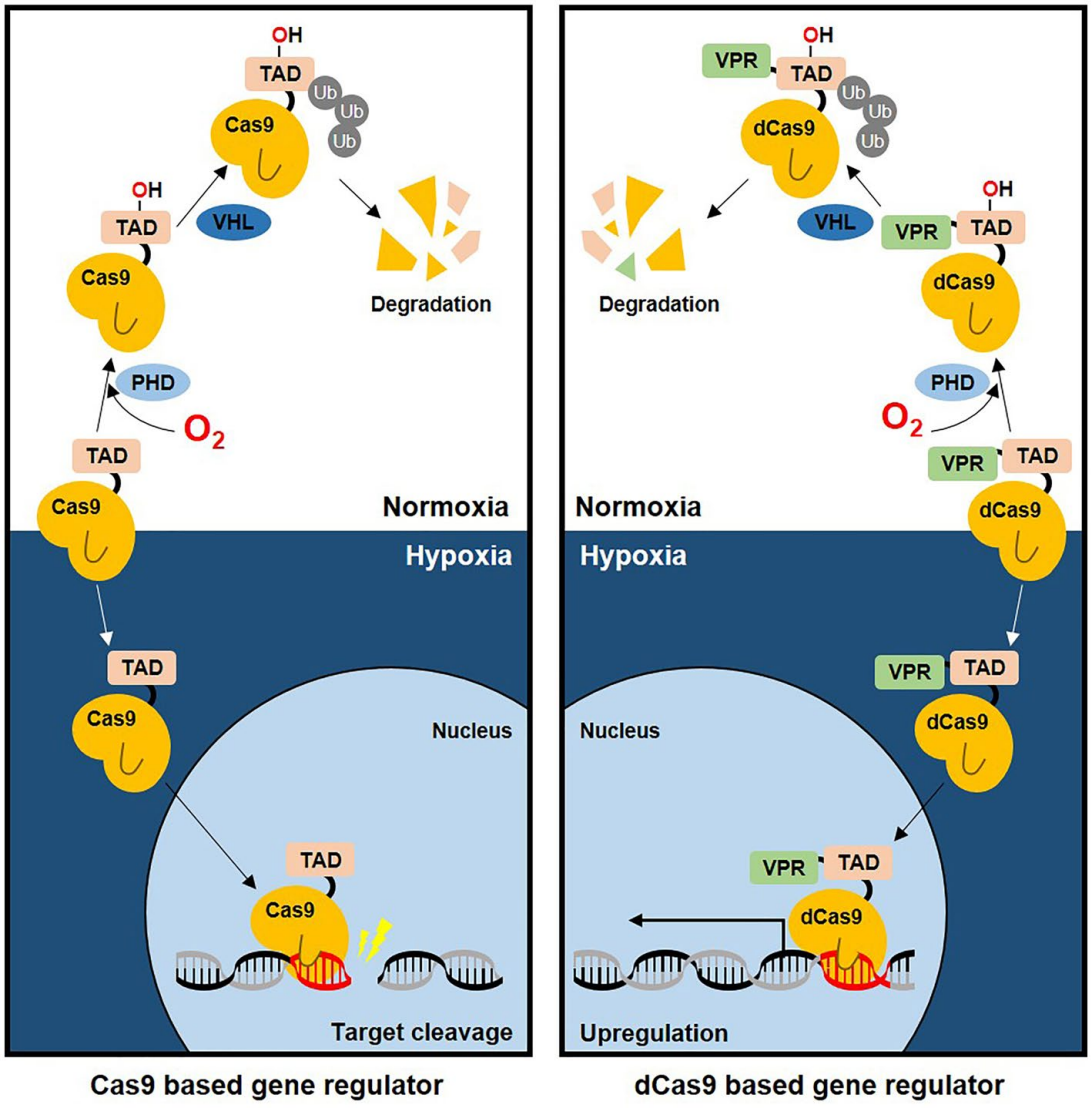
Working diagram of the function of hypoxia-dependent Cas9/dCas9 regulators. Graphical representation of the proposed working model of the function of hypoxia-dependent Cas9/dCas9 gene regulators.

In this study, we developed a strategy to control the activity of CRISPR–Cas9 in response to internal stimuli. This strategy is different from the well-established mechanism of conditional CRISPR–Cas9 regulation by external stimuli^6,10,14^. By combining the specific DNA-targeting activity of CRISPR–Cas9 and the hypoxia-sensing ability of the TAD of HIF-1α, we developed a tool that is capable of modulating gene expression in a hypoxia-dependent manner. Based on prior studies of the HIF1α protein, we propose a model to explain the activity of the Cas9/dCas9 gene expression regulatory system^26^. Under normoxic conditions, the TAD domain, which is fused to the Cas9/dCas9 protein, is marked for ubiquitin-mediated proteasomal degradation by the enzymes PHD and VHL, resulting in the degradation of the conjugate protein. However, under hypoxic conditions, the TAD domain is rescued from degradation, and the conjugate protein is capable of acting on its target sites, where the Cas9 cleaves the target gene and the dCas9 upregulates target gene expression (Fig. 6).

We have conducted a series of experiments to validate our approach. Specifically, we employed CoCl_2_, a well-known agent that mimics hypoxic conditions, to demonstrate successful functional regulation of both Cas9 and dCas9. The temporal effects of CoCl_2_ replicate natural hypoxic conditions, impacting cellular metabolism, transcription regulation, and signaling pathways^54^. Concentration of CoCl_2_ used this study (150uM) also falls within the optimal range (100–300 μM)^54,55^. Through representative experiments, we confirmed that the hypoxia-dependent modulation facilitated by the TAD domain extends to genuine hypoxic conditions as well (Supplementary Figs. S2b, S8c,d). To accurately simulate true hypoxia, we utilized a hypoxic chamber set to 1% O_2_. This level of oxygen closely aligns with the oxygen concentrations found in tumor hypoxia, where levels can be below 2%, and even as low as 0.1% in radiobiological hypoxia^56^. These conditions are strongly associated with radiotherapy resistance. Since our system can induce hypoxia-dependent changes in gene editing efficacy of Cas9/dCas9 at these oxygen levels, it holds promise for efficiently targeting cancer cells within similar or more severe hypoxic environments. Previous studies have shown the stabilization of the HIF/ODD protein at oxygen concentrations relevant to cancer cell lines such as HeLa and U-251MG, in both true hypoxic as well as chemical-induced (BAY-85 3934) hypoxic conditions with comparable results^57^. It is to be noted that BAY-85 3934 mimicks hypoxia through a mechanism similar to CoCl_2_—they both inhibit the activity of PHD^58^. Furthermore, prior studies have used CRISPR-Cas9 for genome wide screen across varying oxygen levels (from 21 to 1%) with no apparent change in efficiency^59^. This supports our confidence in the adaptability of our system to true hypoxic conditions of cancer cells. Our results are particularly compelling. We achieved hypoxia-dependent cleavage of SNAIL, a protein pivotal in cancer metastasis, using the Cas9-TAD. Additionally, dCas9-TAD-VPR led to the up-regulation of tumor-related genes like NEUROD1 and HNF4, all in response to hypoxia. This underscores the potential of our approach in the realm of cancer therapy.

Cas9-TAD and dCas9-TAD did, however, show a certain level of activity (leakiness) under normoxic conditions as well, possibly due to incomplete degradation. This nonspecific activity can be controlled by combining our system with additional hypoxia-inducible systems. For example, expressing the Cas9/dCas9 gene regulators under the control of a promoter previously established to be regulated by hypoxia, the activity of which can be induced by natural hypoxia as well as hypoxia mimetics such as Co^2+^, provides an additional layer of regulation to the system^60^. With this approach, the expression of the Cas9/dCas9 gene regulators could be controlled at the transcriptional level via hypoxia-inducible promoters as well as at the posttranscriptional level via the TAD domain, thereby reducing undesired activity under normoxic conditions. Alternatively, it is possible to include delivery systems that release the Cas9 and dCas9 gene regulators in response to hypoxia. For instance, hypoxia-responsive gold nanorod (AuNR)-based CRISPR–Cas9 nanocomposites release Cas9 in a hypoxia-dependent manner^61^. Such approaches provide the possibility of hypoxia-dependent cargo delivery, and these materials could be incorporated into our design.

In our study, the ability of the dCas9 gene regulator to activate gene expression varied between target genes. It is likely that the inherent stochastic nature of gene expression and epigenetic barriers are responsible for these differences. Previously, to overcome such limitations, alternative approaches, such as improved transactivation domains or catalytically active domains that affect epigenetic states, have been used^62,63^. Additionally, based on our results and previous studies, we propose that increasing the number of gRNAs specific for a particular target gene and using multiple transactivator domains (VPR) can also help enhance target gene expression.

Due to their accurate reflection of many key aspects of human cancer, tumour xenograft models are considered suitable models for in vivo studies of tumours^64^. The effect of CRISPR–Cas9-mediated gene editing on cancer progression is often monitored and studied in xenograft models^64,65^. Hence, to investigate and further validate the potential of our system to be used as a gene therapeutic agent, local injections of viral particles harbouring the TAD-Cas9 or dCas9-TAD-VPR genes and guide RNA cassettes into a tumour xenograft model would be necessary. Additionally, cancer organoid models that exhibit hypoxic conditions could offer an alternative validation platform for this purpose^64^. Alternatively, replacing Cas9 with some of the recently developed mini-CRISPR proteins, such as Cas12f^66^, or transposon-associated RNA-guided endonucleases, such as TnpB, while maintaining the hypoxia-dependent function of the TAD^67^ is worthy of further investigation to attempt to broaden the application prospects of this system.

In addition to cancer, hypoxia is prevalent in many clinically relevant conditions, such as acute mountain sickness, high altitude pulmonary edema, high altitude cerebral edema, and muscle wasting (skeletal muscle loss), in humans^68–70^. With further studies, it may be possible to use hypoxia-sensitive Cas9/dCas9-based gene regulatory systems for the treatment of the conditions mentioned above. Additionally, our system may be applicable in biological hypoxia imaging^71^ and can be used as a high-throughput CRISPR–Cas9 library screening^72^ tool in the fields of functional genomics and drug development to identify genes related to hypoxia.

In conclusion, this proof-of-concept study investigated the conditional regulation of CRISPR–Cas9 in response to the cellular microenvironment. We believe that this regulatory approach could be a novel addition to the CRISPR–Cas9 tool kit.

## Materials and methods

### Cell culture and transfection

The human embryonic kidney cell line HEK293 was used for all the experiments in this study unless otherwise mentioned. HEK293 cell line used in this study was a gift from Dr. Yong-Sam Kim, Korean Research Institute of Bioscience and Biotechnology, Daejeon, Republic of Korea. HEK293 cells were cultured at 37 °C in 5% CO_2_ in Dulbecco’s modified Eagle’s medium (Gibco) supplemented with 10% (v/v) foetal bovine serum (FBS; HyClone). The cells were dissociated with trypsin after reaching 80–90% confluence. All the plasmids used for transfection were obtained with the Midi Prep using a Plasmid Midi kit (25) (Qiagen®). Transfections were carried out in six-well plates using LipofectamineTM 2000 Transfection Reagent and FuGENE® HD Transfection Reagent. 2 µl of LipofectamineTM 2000 reagent was used per 1 µg DNA, and 3 µl of FuGENE reagent was used per 1 µg DNA. When necessary, the cells were treated with 150 µM CoCl_2_ to induce hypoxia. To simulate a true hypoxic environment for cell culture experiments, a specialized hypoxic chamber was utilized, which was stabilized at 1% O_2_.

### Generation of cell line

A HEK293 cell line stably expressing the luciferase gene in its genomic DNA was generated using X-treme GENE HP DNA transfection reagent. The linear gal4 pGL4.21-Gal4-Luc sequence was transfected into HEK293 cells and grown in DMEM (Gibco) supplemented with 10% (v/v) FBS. After 48 h of transfection, puromycin was added to the medium to a final concentration of 1 µg/ml, and the culture was further incubated for two weeks. The obtained colonies were separated into 24-well plates and further cultured for three weeks. Finally, the Gal-TAD fusion protein was used to verify the successful insertion of the luciferase gene into the genomic DNA.

### Construction of plasmids

The plasmid pCMV-dxCas (3.7)-VPR (gift from Dr. David Liu, Addgene #108383) was used to construct all three variants of the dCas9-VPR-TAD fusion protein. pCMV-TAD-dCas9-VPR was generated by introducing the AgeI restriction enzyme site upstream of dCas9 through site-directed mutagenesis (SDM) and inserting the TAD domain. Similarly, pCMV-dCas9-TAD-VPR and pCMV-dCas9-VPR-TAD were generated by introducing the NheI and HpaI restriction sites, respectively. All the TAD-Cas9 fusion protein variants were generated by modifying the original plasmid pCDNA 3.3-spCas9-VPR. Through SDM PCR, a stop codon was introduced upstream of VPR to prevent VPR expression. To generate TAD-Cas9, the restriction site AgeI was introduced upstream of Cas9, and the TAD was inserted at this site. Similarly, TAD was introduced downstream of Cas9 at the NheI site. The TAD-Cas9-TAD sandwich protein was created by inserting an additional TAD upstream of Cas9 into the pCDNA-Cas9-TAD construct at the NotI restriction site.

### Western blotting analysis

For the western blot assays, cells were cultured under normoxic conditions for 24 h following transfection. Subsequently, the cells were either treated with CoCl_2_ or subjected to hypoxic conditions for 12 h before being harvested. Cells were lysed in 1X passive lysis buffer (Promega) in the presence of a protease inhibitor cocktail (cOmplete mini). The proteins were separated by 12% SDS–PAGE and transferred to Hybond ECL membranes (GE healthcare) or Amersham Protran Premium 0.45 NC nitrocellulose western blotting membranes. Nonspecific binding was blocked using 5% skim milk in 1X TBST. The blots were probed with primary monoclonal mouse anti-Cas9 antibodies (Santa Cruz) or Monoclonal ANTI-FLAG^®^ M2 antibodies (Sigma–Aldrich) for 2 h, and similarly, the control was probed with antibodies against α-tubulin (Sigma). The blots were washed with 1X TBST 5 times at 5 min intervals. The blots were further probed with mouse secondary antibodies (Jackson ImmunoResearch) for 1 h. The membranes were washed 5 times with 1× TBST as mentioned before. The blots were developed using ClarityTM western ECL substrate reagent (Bio-Rad) and imaged using a TM 300E instrument and Amersham Imager 680. Unless stated otherwise, all blots were bisected to facilitate the separation of conjugate proteins and the positive control (α-Tubulin). Subsequently, the hybridization step was performed individually with their corresponding primary antibodies.

### Luciferase assay

The reagents for the assay in which the luciferase gene was cleaved by Cas9 were used at the following concentrations: 400 ng firefly luciferase plasmid (pCDNA3), 100 ng pRL-TK Renilla luciferase plasmid, 1.5 µg Cas9 and Cas9 conjugates plasmid, and 0.9 µg sgRNA. For the assay in which the luciferase gene transcription was activated by dCas9, the reagents were used at final concentrations as follows: 400 ng firefly luciferase plasmid (4 gal4-binding sites), 100 ng pRL-TK Renilla luciferase plasmid, 2 µg dCas9 and dCas9 conjugates plasmid, and 0.5 µg sgRNA specific for luciferase and expressed on plasmid; 100 ng pRL-TK Renilla luciferase plasmid, 2 µg dCas9 and conjugates plasmid, and 0.5 µg sgRNA specific for luciferase and expressed in the genome of the stable cell line. A luciferase reporter assay was performed using a Dual-Luciferase® Reporter Assay kit (Promega). Cells were lysed in 1X lysis buffer (5× passive lysis buffer, Promega) in the presence of a protease inhibitor (cOmplete Mini) for 30 min under shaking conditions. 100 µl of Luciferase assay reagent II was prepared by resuspending the luciferase assay substrate in Luciferase assay buffer II. Stop & Glo reagent was prepared by resuspending the Stop & Glo substrate in Stop & Glo buffer. 20 µl of the cell lysate was mixed with 100 µl of luciferase assay reagent II at room temperature, and the firefly luciferase activity was measured using a Multimode Plate Reader VICTORTM × 3 instrument (PerkinElmer). Additionally, 100 µl of Stop & Glo reagent was added at room temperature, and the Renilla luciferase activity was recorded.

### mRFP-eGFP reporter assay

Cas9/Cas9-TAD plasmid (1.5 µg), sgRNA (0.9 µg), and mRFP-eGFP plasmid (400 ng) were used. After 48 h of transfection, the RFP and GFP expression of cells grown in 6-well plates was measured using an EVOS FL AUTO2 NO OBJ/NO CUBE EACH (Thermo) instrument.

### T7 endonuclease assay

After 48 h of transfection and hypoxia treatment, cells were harvested, and genomic DNA was extracted using the PureHelix™ Genomic DNA Prep kit. The target locus was amplified using an AccuPower® PCR PreMix kit (Bioneer). The obtained product was denatured by incubating at 95 °C for 5 min and reannealed by decreasing the temperature at a rate of 1 °C/1 min until it reached room temperature. Five microlitres of the sample was mixed with 1 µl of 10× buffer and 0.5 µl of T7 endonuclease I enzyme (NEB) and incubated for 1 h at 37 °C. The product was resolved on a 10% TBE gel, and staining was performed using SYBR™ Safe DNA Gel Stain (Invitrogen). Then, the staining was visualized with a Gel doc and quantified using ImageJ software.

### NGS sequencing

1.5 µg Cas9 and Cas9 conjugates plasmid, and 0.9 µg sgRNA were used for the assay. Genomic DNA was extracted using the PureHelix™ Genomic DNA Prep kit. The first round of PCR was performed using adapter primers and 100 ng of the extracted DNA. The second round of PCR was performed on the products from the first round of PCR using index primers. All the PCRs were performed using an AccuPower® PCR PreMix kit (Bioneer). The PCR products were resolved on 2% agarose gel. The target bands were extracted from the gel using a DNA, RNA, and protein purification kit (Macherey–Nagel™). Extracted DNA at a concentration of 15 ng/µl was sent for NGS sequencing where sequencing was done using Illumina iSeq100 platform.

### qRT–PCR

2 µg dCas9 and dCas9 conjugates plasmid, and 1.2 µg sgRNA were used in the assay. A total of 1000 ng of extracted RNA was used as a template to synthesize cDNA using the RevertAid H Minus First Strand cDNA Synthesis Kit (Thermo).qRT–PCR was performed following the instructions of the TOPreal™ qPCR 2X PreMIX (SYBR Green with low ROX). A total of 1.5 µl of the sample was mixed with 10 µl of 2 × mix buffer and 1 µl of forward and reverse primers, and the volume was adjusted to prepare 20-µl qRT–PCR reactions. The reaction was monitored and analyzed using a CFX96 Real-Time PCR System (Bio-Rad) and CFX Manager software (Bio-Rad). The obtained results were quantified using delta-delta-Ct method and normalized to the expression of GAPDH. Cycle conditions used for qRT-PCR are as follows: initial denaturation at 95 °C for 15 min, denaturation at 95 °C for 10 s, annealing at 60 °C for 15 s, elongation at 72 °C for 30 s, number of cycles 35.

### Statistical analysis

Statistical analysis was performed by t-test: paired two sample for means, using Microsoft Excel 2016 software.

### Supplementary Information

Supplementary Information.

## Data Availability

The data that support the findings of this study are available in the methods and/or supplementary material of this article.
