# Supplementary material for: Design of hypoxia responsive CRISPR-Cas9 for target gene regulation
Source: Sci Rep. 2023 Oct 5;13:16763. doi: 10.1038/s41598-023-43711-9 (PMC10556097; doi:10.1038/s41598-023-43711-9)
Supplement: Supplementary file 1 — Supplementary Information. [file 41598_2023_43711_MOESM1_ESM.pdf]

Supplementary Information

Design of hypoxia responsive CRISPR-Cas9 for target gene regulation

Yan An[1,3*], Chandana S.Talwar[1,2*], Kwang-Hyun Park[1], Woo-Chan Ahn[1], Su-Jin Lee[1,2] Seong-Ryeong Go[1,2], Jin Hwa Cho[1], Do Yon Kim[1,2], Yong-Sam Kim[1,2], Sayeon Cho[4], Jeong-Hoon Kim[1,2], Tae-Jip Kim[3], Eui-Jeon Woo[*1,2]

1 Division of Biomedical research,
Korea Research Institute of Bioscience and Biotechnology,
Daejeon, 305-333, Republic of Korea.

2 Department of Bioscience,
University of Science and Technology,
Daejeon, 305-333, Republic of Korea.

3 Division of Animal, Horticultural and Food Sciences University of Science and Technology,
Graduate School of Chungbuk National University,
Cheongju, 28644, Republic of Korea.

4 Laboratory of Molecular and Pharmacological Cell Biology,
College of Pharmacy,
Chung-Ang University,
Seoul, 06974 Republic of Korea.

Correspondence should be addressed to E.W. (E-mail: ejwoo@kribb.re.kr)

Short title: Hypoxia-dependent CRISPR-Cas9 for gene regulation

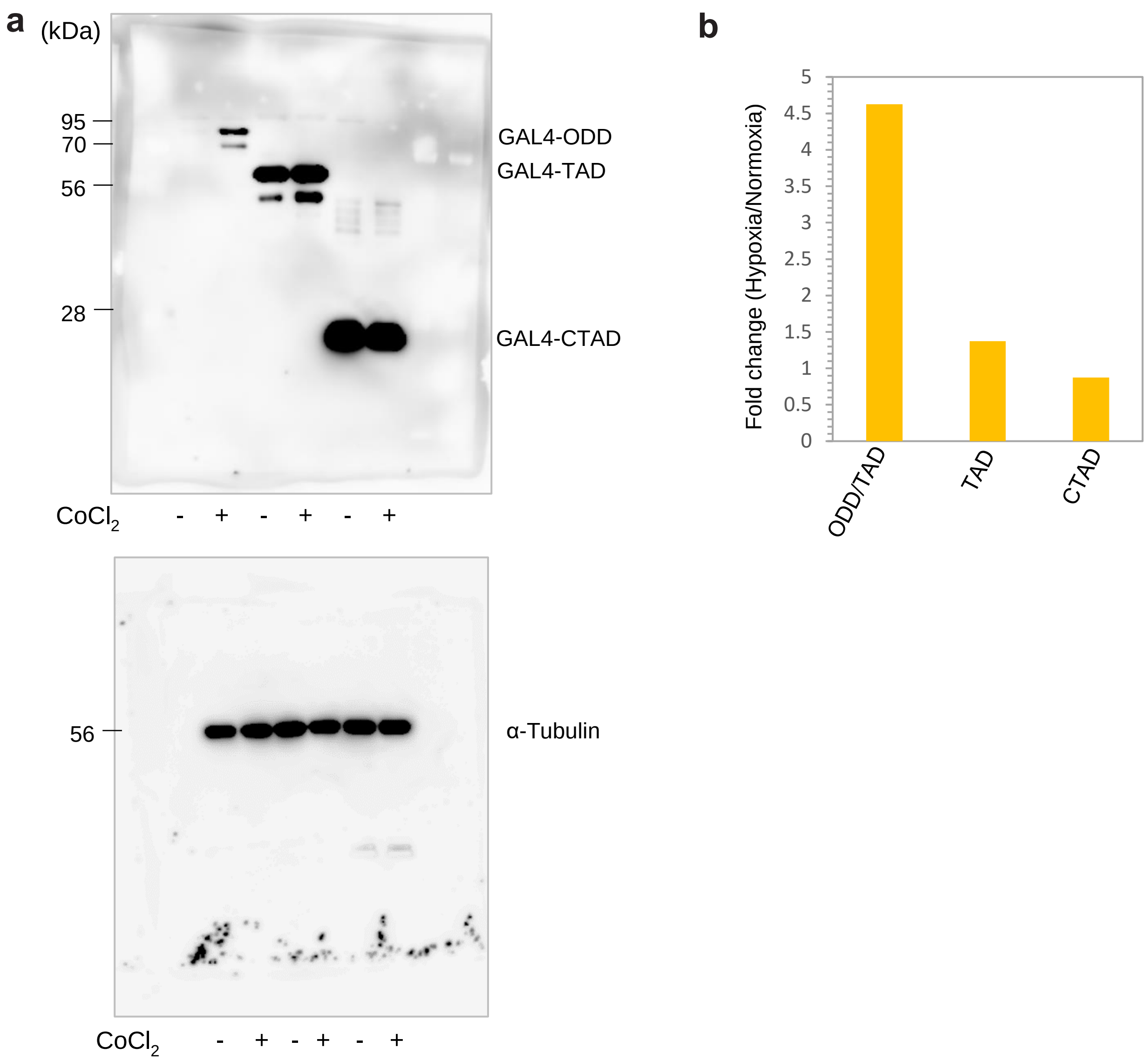

Supplementary Fig. 1: (a) Western blotting results showing the protein expression of the truncated variants of HIF-1α under normoxic and hypoxic conditions. The lower lane shows the protein level of α-Tubulin, which was used as a control. α-Tubulin blot was processed separately on a different blot due to the overlap in size between target proteins and α-Tubulin. (b) Intensity of the western blotting bands as quantified by ImageJ software.

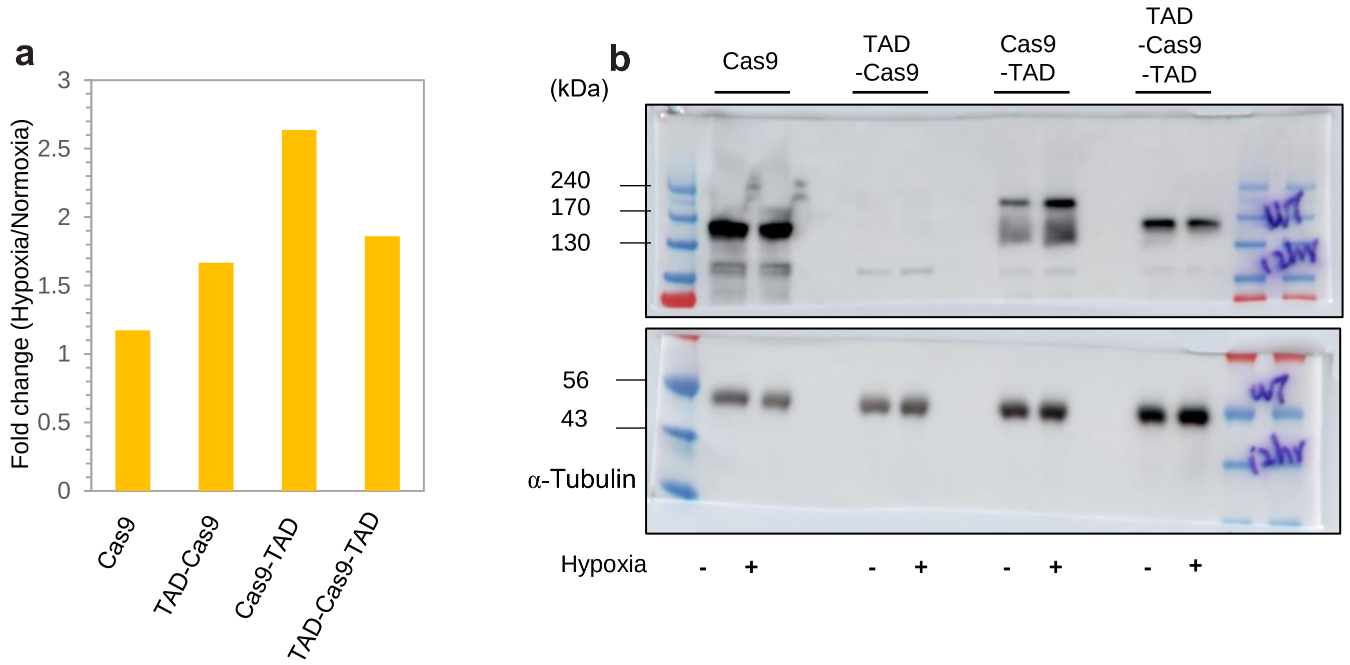

Supplementary Fig. 2: (a) Quantified western blotting results showing the protein levels of the conjugate proteins under normoxic and hypoxic conditions. (b) Western blotting image showing the effect of TAD conjugation on Cas9 protein in true hypoxia (1% O_2) as induced using hypoxic chamber. Upper panel shows Cas9-TAD conjugate proteins and lower panels shows α-Tubulin respectively. Proteins are probed using anti-Cas9 antibody and anti-Tubulin antibody after 12 hrs of hypoxic treatment.

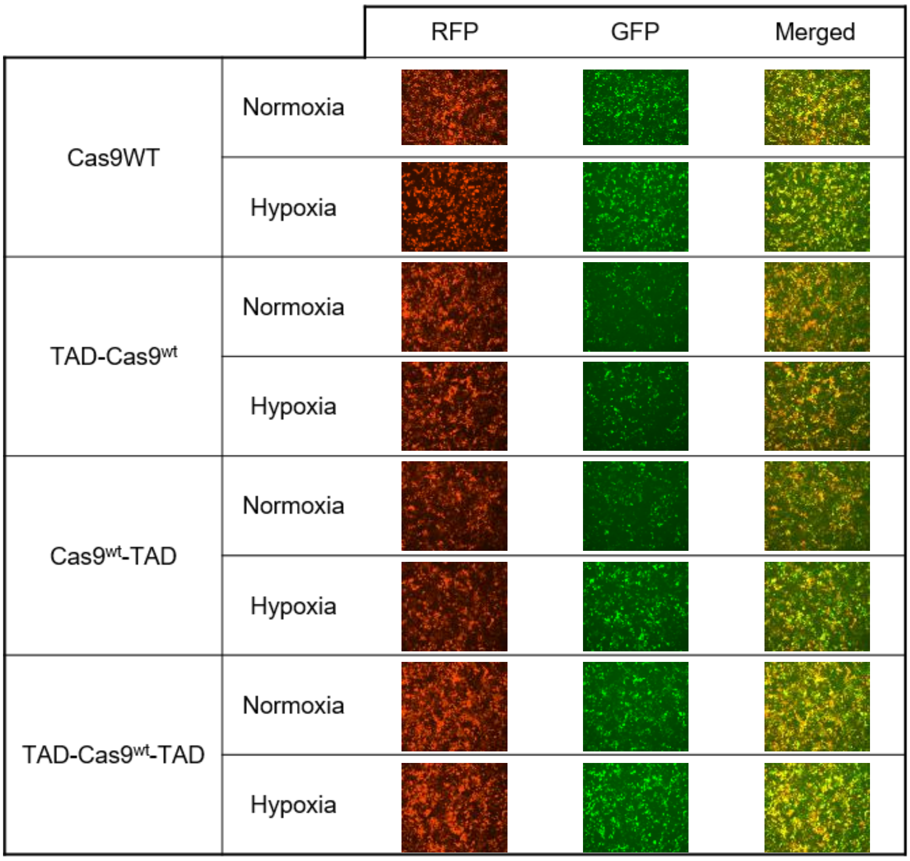

Supplementary Fig. 3: mRFP-eGFP reporter assay showing the hypoxia-dependent activity of the Cas9 and TAD conjugate proteins. RFP, GFP and merged images are shown for each of the conjugate proteins

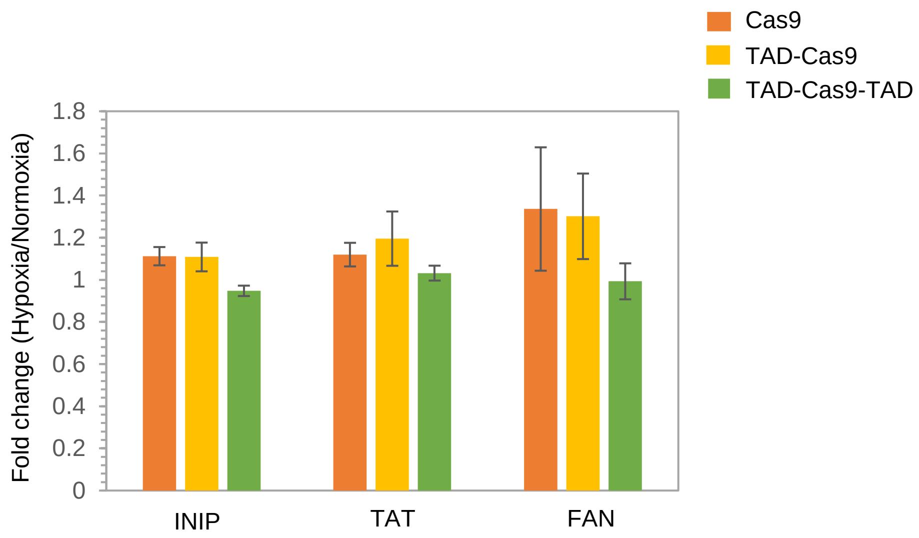

Supplementary Fig. 4: Hypoxia-dependent activity of TAD-Cas9 and TAD-Cas9-TAD on the endogenous genes INIP, FAN and TAT, as shown by NGS sequencing. The bars in orange, yellow and green represent Cas9, TAD-Cas9 and TAD-Cas9-TAD, respectively. The error bars were calculated from the results of three replicates.

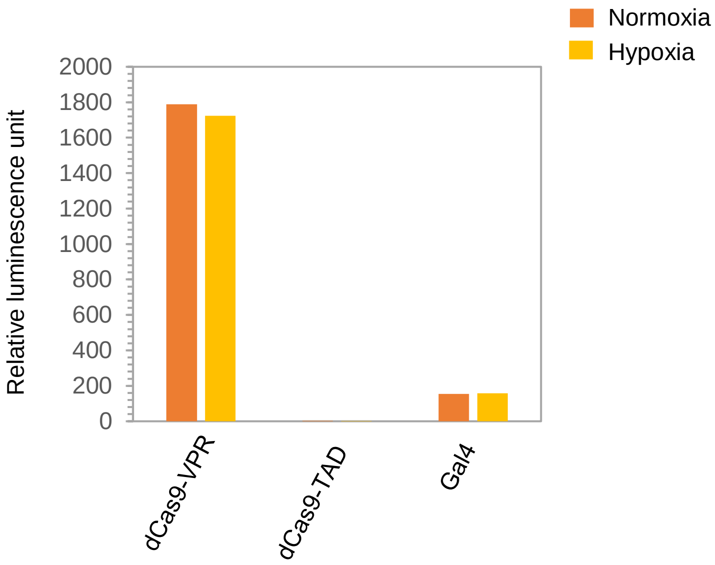

Supplementary Fig. 5: Luciferase reporter assay showing transcriptional activation by dCas9-VPR, dCas9-TAD and Gal4. Transcriptional activation of a luciferase gene located downstream of UAS in a plasmid by dCas9-TAD and dCas9-VPR. The gene was targeted using sgRNA. The control luciferase gene was also targeted using a Gal4-binding domain.

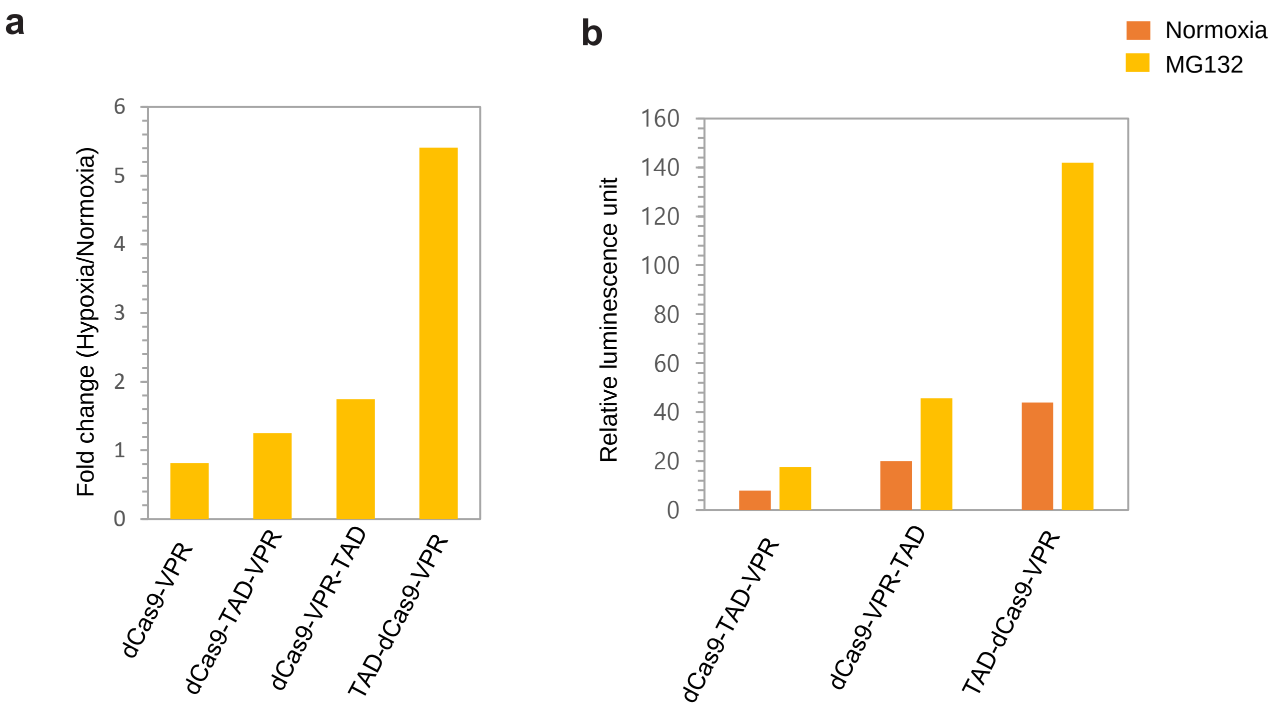

Supplementary Fig. 6: (a) Quantified western blotting results showing the protein levels of the conjugate proteins under normoxic and hypoxic conditions. (b) Effect of the proteasome inhibitor MG-132 as shown by a luciferase reporter assay. Both cultures were grown under standard growth conditions (normoxia) in the presence or absence of MG-132.

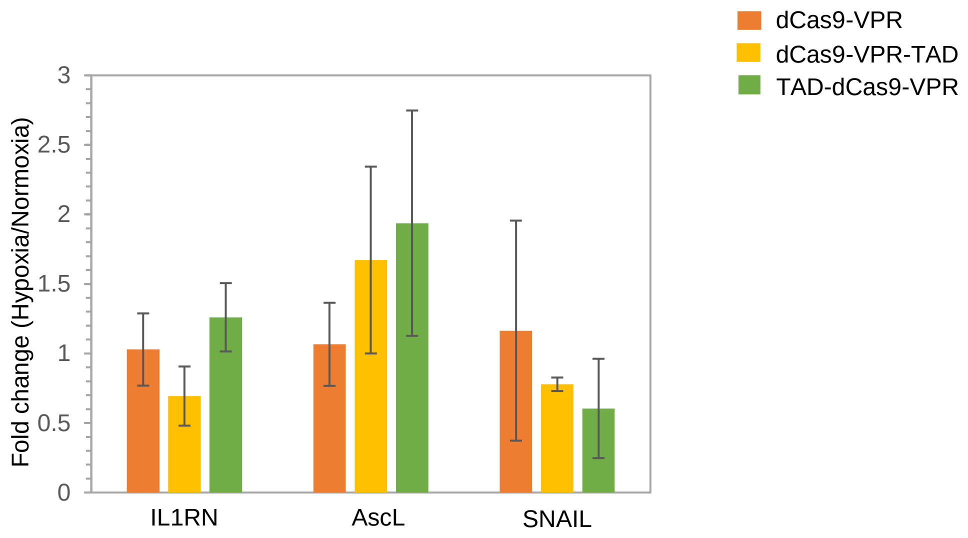

Supplementary Fig. 7: Activity of dCas9-VPR-TAD and TAD-dCas9-VPR on the endogenous genes IL1RN, AscL1 and SNAIL as shown by qPCR.

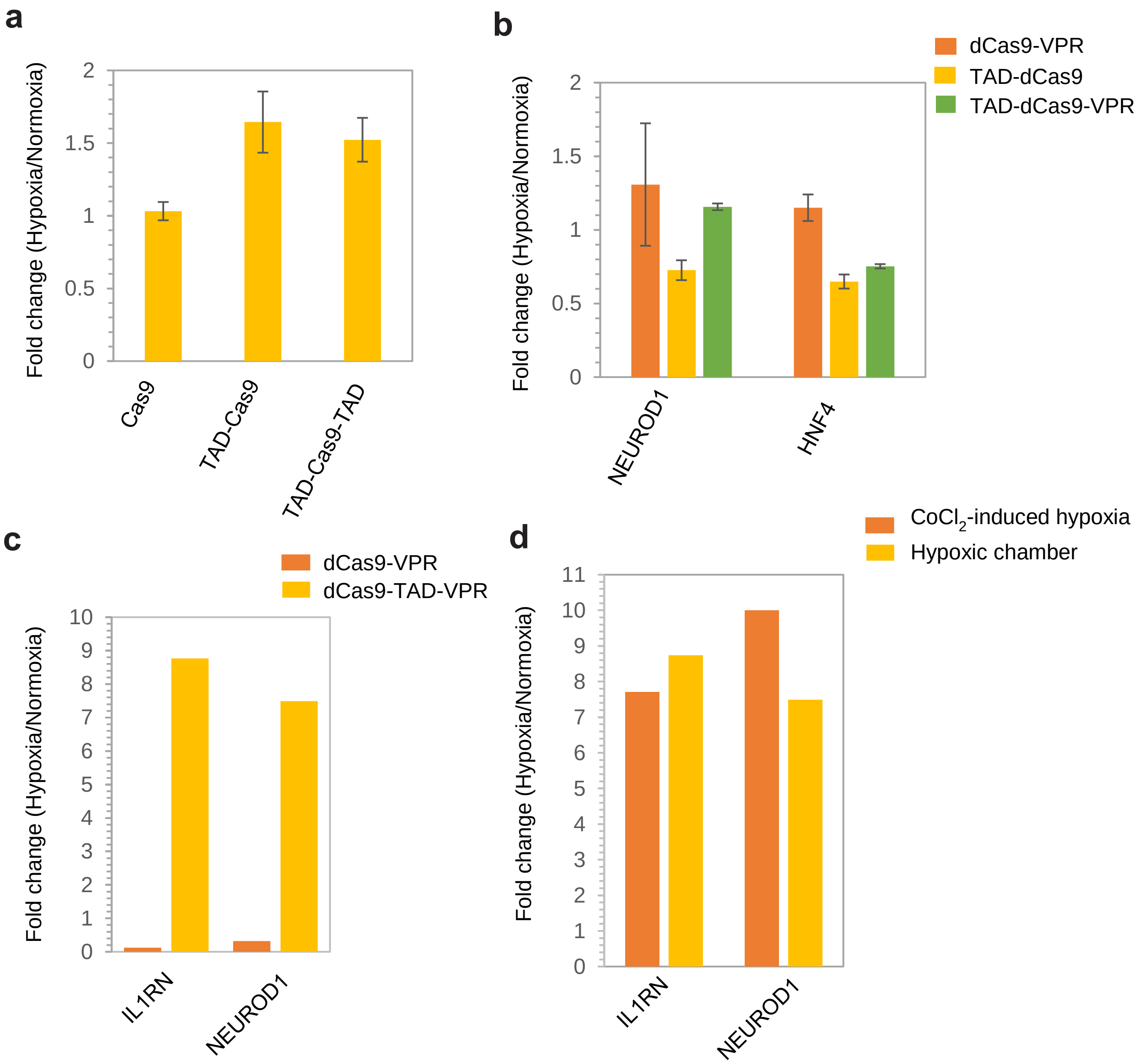

Supplementary Fig. 8: (a) Hypoxia-dependent downregulation of the expression of the endogenous gene SNAIL using TAD-Cas9 and TAD-Cas9-TAD, as shown by NGS sequencing. (b) Hypoxia-dependent upregulation of the expression of the endogenous genes NEUROD1 and HNF4 using dCas9-VPR-TAD and TAD-dCas9-VPR. (c) dCas9-VPR and dCas9-TAD-VPR were targeted to IL1RN and NEUROD1 using respective sgRNAs. Their upregulation was quantified using qPCR after 48 hrs of exposure to hypoxia in hypoxic chamber. Orange bar and yellow bars shows the fold change in expressions as targeted by dCas9-VPR and dCas9-TAD-VPR respectively for both IL1RN and NEUROD1. (d) This graph compares the fold change in expression of IL1RN and NEUROD1 brought by dCas9-TAD-VPR in $CoCl_2$ induced hypoxic condition and exposure to hypoxia in hypoxic chamber.

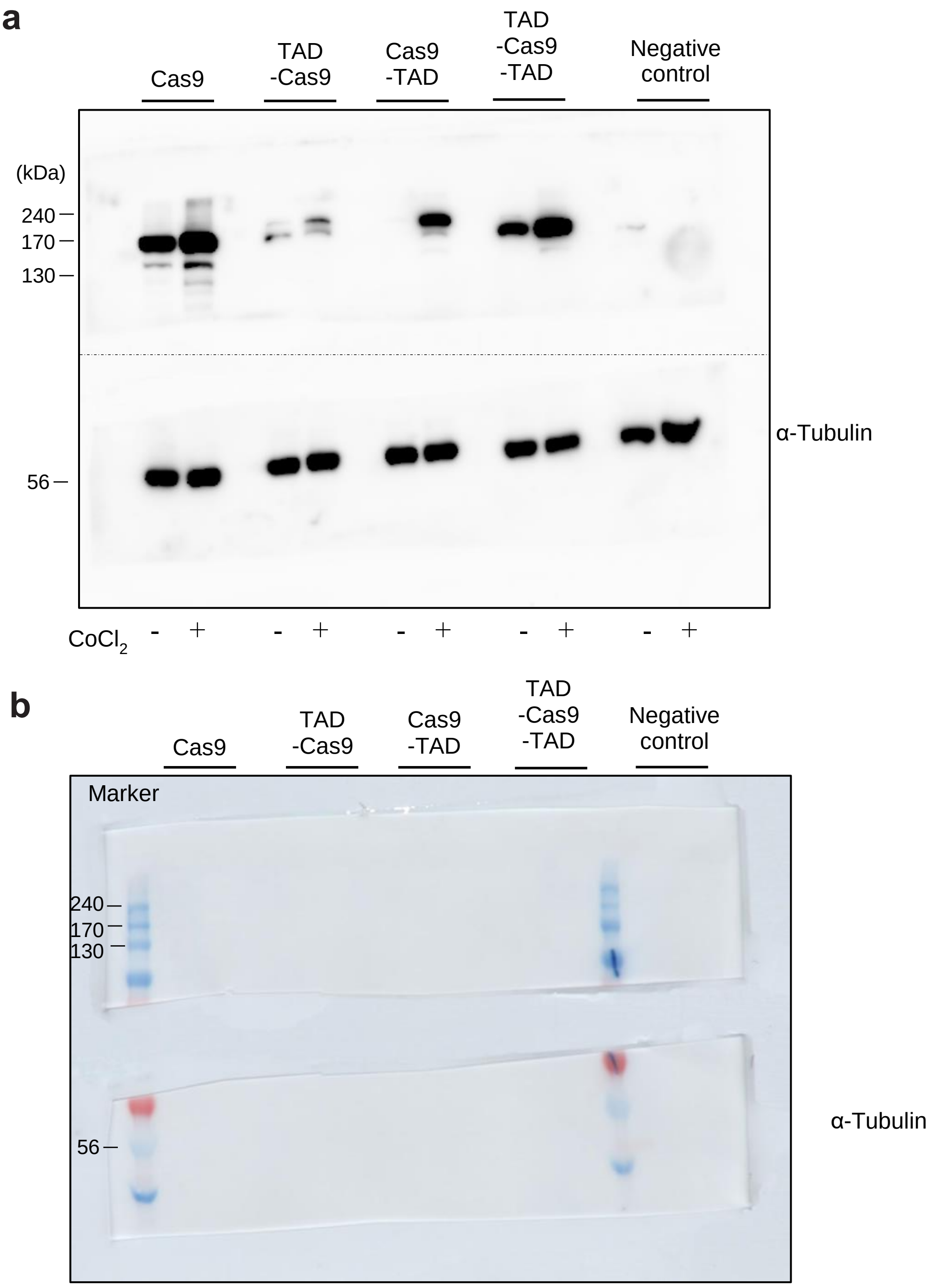

Supplementary Fig. 9: Complete image of Fig. 2b as obtained from the experiment. (a) Chemiluminescent image showing the expression of Cas9 and its conjugates in the upper panel and tubulin expression in the lower panel. Negative control refers to cell samples where transfection was conducted with empty vector which does not carry any of the Cas9 variants. (b) Image of the blot showing the marker bands.

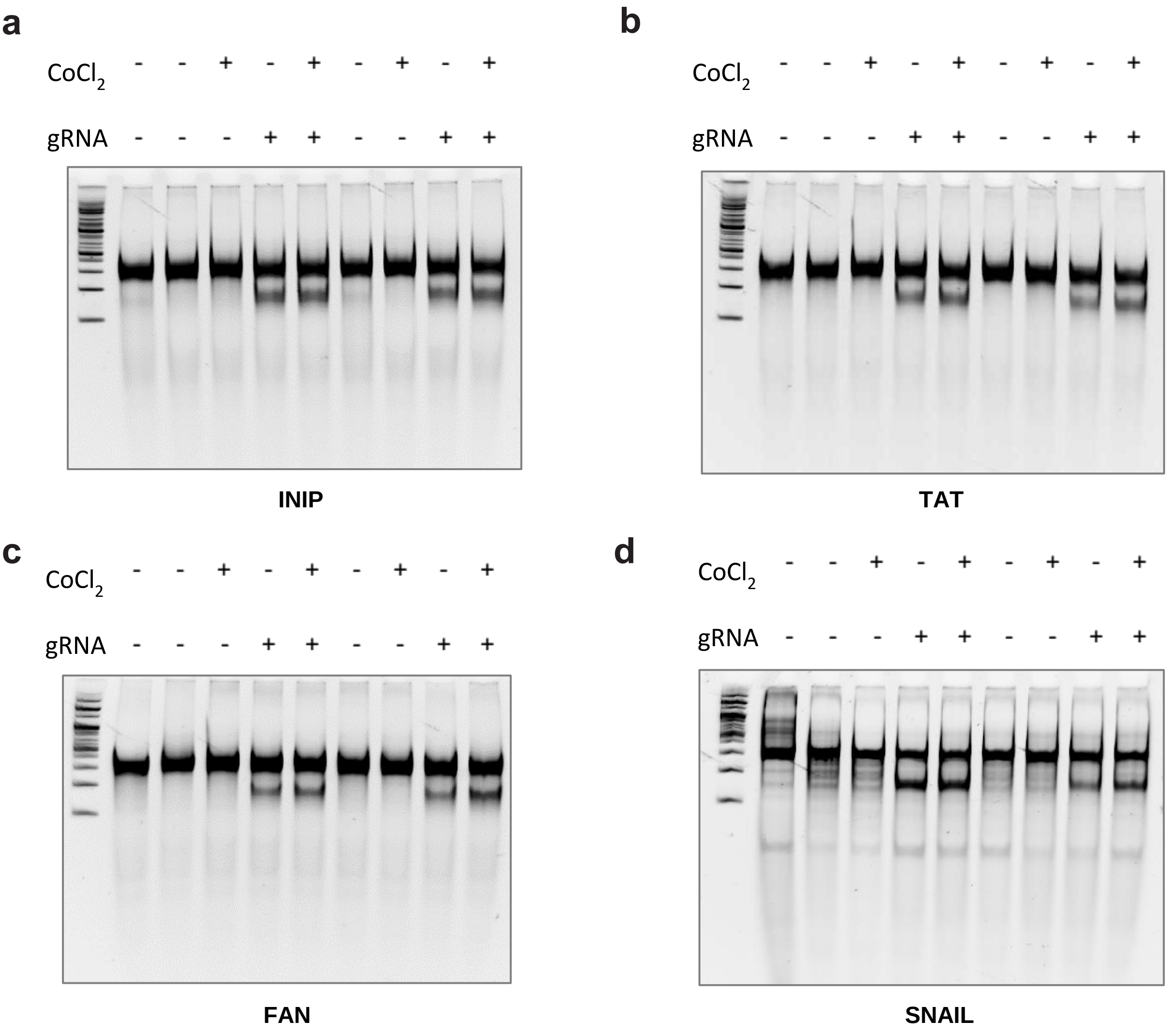

Supplementary Fig. 10: Full gel images of Fig .3b, 3c , 3d and 5a. Cas9-TAD activity on the endogenous genes (a) INIP, (b) TAT, (c) FAN, and (d) SNAIL as shown by the T7 endonuclease assay.

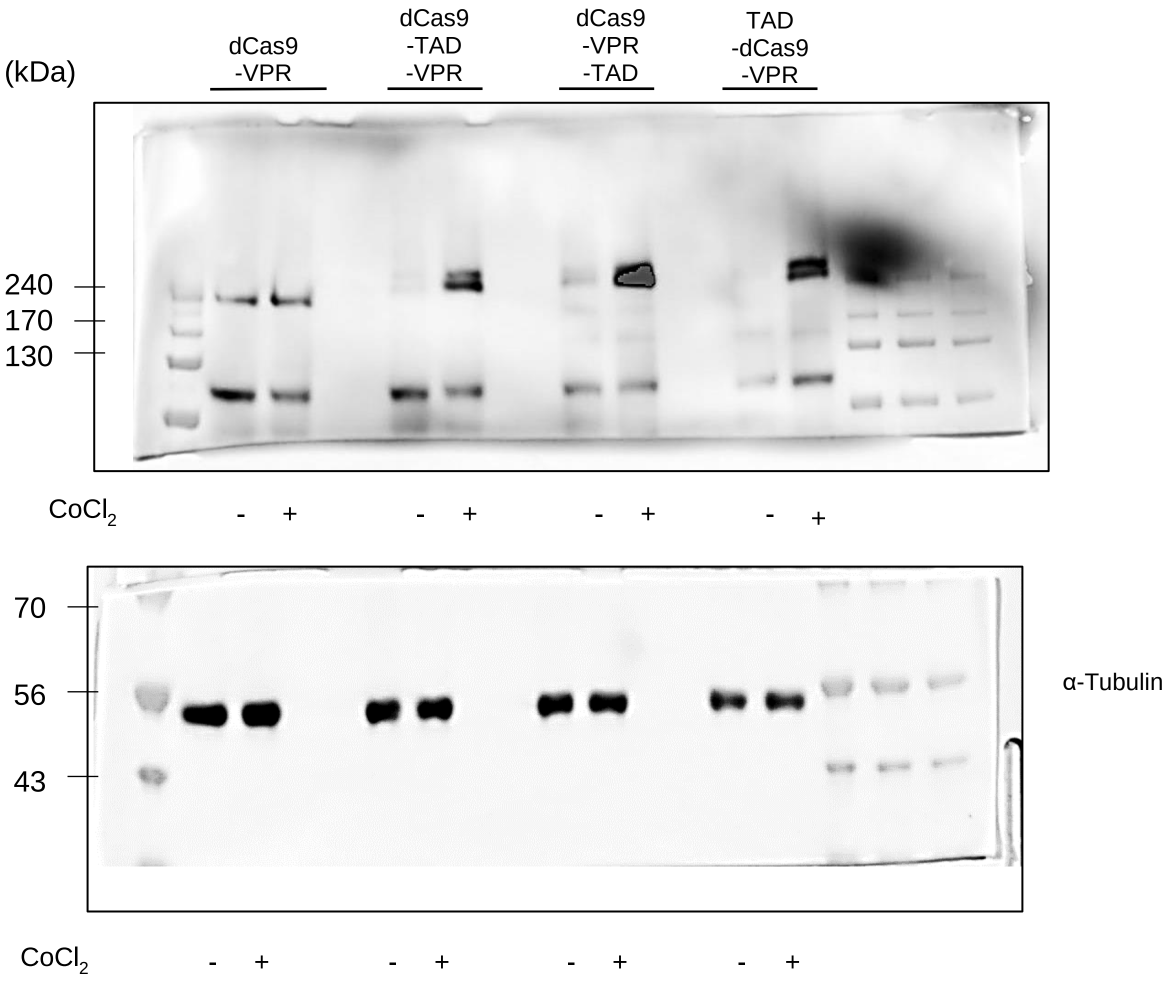

Supplementary Fig. 11: Unedited western blot image of Fig. 4b as obtained from the experiment. Upper panel shows the protein expression of dCas9 conjugates and lower panel shows α-Tubulin expression under normoxic and hypoxic conditions. Hybridization step was conducted separately for dCas9 conjugate proteins and α-Tubulin with respective primary antibodies.

Table S1: Target sites used in this study.

Target	Sequence
TAT	TCCTCCTGAGACTCCATACC
INIP	GCAGCGATTGTAAGGAGAGG
FANCF	GGAATCCCTTCTGCAGCACC
SNAIL	GTTAGGCTTCCGATTGGGGT
IL1RN	TGTACTCTCTGAGGTGCTC
	ACGCAGATAAGAACCAGTT
	CATCAAGTCAGCCATCAGC
	GAGTCACCCTCCTGGAAAC
AscL1	CGGGAGAAAGGAACGGGAGG
	AAGAACTTGAAGCAAAGCGC
	TCCAATTTCTAGGGTCACCG
	GTTGTGAGCCGTCCTGTAGG
NEUROD1	AGGGGAGCGGTTGTCGGAGG
	ACCTGCCCATTTGTATGCCG
	AGGTCCGCGGAGTCTCTAAC
	TAGAGGGGCCGACGGAGATT
SNAIL (inducible)	CCGAAGGCCACTCCCCGAGC
	CGAGCCTCCGATTGGCGCGG
HNF4	CCAGTCACTTAGGGAACCCG
Luciferase	GATGCCAAAAACATTAAGAA
GFP/RFP	TGACATCAATTATTATACATCGG
Gal4 binding site	CGGAGTACTGTCCTCCGAG

Table S2: PCR primers used for T7 endonuclease and NGS sequencing

Target	primer	
TAT	Forward	ACACTCTTTCCCTACACGACGCTCTTCCGATCTTACAGACCCTGAAGTTACCC
	Reverse	GTGACTGGAGTTCAGACGTGTGCTCTTCCGATCTGGACCATGTAATCTTAGCCT
FANCF	Forward	ACACTCTTTCCCTACACGACGCTCTTCCGATCTTTGCAGAGAGGCGTATCATT
	Reverse	GTGACTGGAGTTCAGACGTGTGCTCTTCCGATCTTGGATGTGGCGCAGGTAGCG
INIP	Forward	ACACTCTTTCCCTACACGACGCTCTTCCGATCTAGCAACAAAGACGGCGGCGA
	Reverse	GTGACTGGAGTTCAGACGTGTGCTCTTCCGATCTACGTCAGTCAGGAGGCGGAA
SNAIL	Forward	ACACTCTTTCCCTACACGACGCTCTTCCGATCTCCCCACGCAGCCTTCGCCTG
	Reverse	GTGACTGGAGTTCAGACGTGTGCTCTTCCGATCTTAGCGAGTGGTTCTTCTGCG

Table S3: NGS index primers used in this study

**NGS index sequence**	
D501	AATGATACGGCGACCACCGAGATCTACACTATAGCCTACACTCTTTCCCTACACGAC
D502	AATGATACGGCGACCACCGAGATCTACACATAGAGGCACACTCTTTCCCTACACGAC
D503	AATGATACGGCGACCACCGAGATCTACACCCTATCCTACACTCTTTCCCTACACGAC
D504	AATGATACGGCGACCACCGAGATCTACACGGCTCTGAACACTCTTTCCCTACACGAC
D505	AATGATACGGCGACCACCGAGATCTACACAGGCGAAGACACTCTTTCCCTACACGAC
D506	AATGATACGGCGACCACCGAGATCTACACTAATCTTAACACTCTTTCCCTACACGAC
D507	AATGATACGGCGACCACCGAGATCTACACCAGGACGTACACTCTTTCCCTACACGAC
D508	AATGATACGGCGACCACCGAGATCTACACGTACTGACACACTCTTTCCCTACACGAC
D701	CAAGCAGAAGACGGCATACGAGATCGAGTAATGTGACTGGAGTTCAGACGTGT
D702	CAAGCAGAAGACGGCATACGAGATTCTCCGGAGTGACTGGAGTTCAGACGTGT
D703	CAAGCAGAAGACGGCATACGAGATAATGAGCGGTGACTGGAGTTCAGACGTGT
D704	CAAGCAGAAGACGGCATACGAGATGGAATCTCGTGACTGGAGTTCAGACGTGT
D705	CAAGCAGAAGACGGCATACGAGATTTCTGAATGTGACTGGAGTTCAGACGTGT
D706	CAAGCAGAAGACGGCATACGAGATACGAATTCGTGACTGGAGTTCAGACGTGT
D707	CAAGCAGAAGACGGCATACGAGATAGCTTCAGGTGACTGGAGTTCAGACGTGT
D708	CAAGCAGAAGACGGCATACGAGATGCGCATTAGTGACTGGAGTTCAGACGTGT
D709	CAAGCAGAAGACGGCATACGAGATCATAGCCGGTGACTGGAGTTCAGACGTGT
D710	CAAGCAGAAGACGGCATACGAGATTTCGCGGAGTGACTGGAGTTCAGACGTGT
D711	CAAGCAGAAGACGGCATACGAGATGCGCGAGAGTGACTGGAGTTCAGACGTGT
D712	CAAGCAGAAGACGGCATACGAGATCTATCGCTGTGACTGGAGTTCAGACGTGT

Table S4: PCR primers used for qRT-PCR.

Target	primer	
AscL	Forward	CGCGGCCAACAAGAAGATG
	Reverse	CGACGAGTAGGATGAGACCG
SNAIL	Forward	TGTCTGCGTGGGTTTTTGTA
	Reverse	GGGGGTGGATTATTGCATAG
IL1RN	Forward	GGA AGA TGT GCC TGT CCT GT
	Reverse	CGC TTG TCC TGC TTT CTG TT
HNF4	Forward	GACATTCGGGCGAAGAAGAT
	Reverse	AAGATGATGGCTTTGAGGTAGG
NEUROD1	Forward	GGATGACGATCAAAAGCCCAA
	Reverse	GCGTCTTAGAATAGCAAGGCA
GAPDH	Forward	GGTATCGTGGAAGGACTCATGAC
	Reverse	ATGCCAGTGAGCTTCCCGTTCAGC
